# CDK8 and CDK19: positive regulators of signal-induced transcription and negative regulators of Mediator complex proteins

**DOI:** 10.1093/nar/gkad538

**Published:** 2023-06-28

**Authors:** Mengqian Chen, Jing Li, Li Zhang, Lili Wang, Chen Cheng, Hao Ji, Serena Altilia, Xiaokai Ding, Guoshuai Cai, Diego Altomare, Michael Shtutman, Stephanie D Byrum, Samuel G Mackintosh, Alexey Feoktistov, Nataliya Soshnikova, Vladislav A Mogila, Victor Tatarskiy, Maksim Erokhin, Darya Chetverina, Angga Prawira, Yi Ni, Stephan Urban, Campbell McInnes, Eugenia V Broude, Igor B Roninson

**Affiliations:** Department of Drug Discovery and Biomedical Sciences, College of Pharmacy, University of South Carolina, Columbia, SC 29208, USA; Senex Biotechnology, Inc. Columbia, SC 29208, USA; Department of Drug Discovery and Biomedical Sciences, College of Pharmacy, University of South Carolina, Columbia, SC 29208, USA; Department of Drug Discovery and Biomedical Sciences, College of Pharmacy, University of South Carolina, Columbia, SC 29208, USA; Department of Drug Discovery and Biomedical Sciences, College of Pharmacy, University of South Carolina, Columbia, SC 29208, USA; Department of Drug Discovery and Biomedical Sciences, College of Pharmacy, University of South Carolina, Columbia, SC 29208, USA; Department of Drug Discovery and Biomedical Sciences, College of Pharmacy, University of South Carolina, Columbia, SC 29208, USA; Department of Drug Discovery and Biomedical Sciences, College of Pharmacy, University of South Carolina, Columbia, SC 29208, USA; Department of Drug Discovery and Biomedical Sciences, College of Pharmacy, University of South Carolina, Columbia, SC 29208, USA; Department of Environmental Health Sciences, Arnold School of Public Health, University of South Carolina, Columbia, SC 29208, USA; Department of Drug Discovery and Biomedical Sciences, College of Pharmacy, University of South Carolina, Columbia, SC 29208, USA; Department of Drug Discovery and Biomedical Sciences, College of Pharmacy, University of South Carolina, Columbia, SC 29208, USA; Department of Biochemistry and Molecular Biology, University of Arkansas for Medical Sciences, Little Rock, AR 72205, USA; Department of Biochemistry and Molecular Biology, University of Arkansas for Medical Sciences, Little Rock, AR 72205, USA; Institute of Gene Biology, Russian Academy of Sciences, 119334 Moscow, Russian Federation; Institute of Gene Biology, Russian Academy of Sciences, 119334 Moscow, Russian Federation; Institute of Gene Biology, Russian Academy of Sciences, 119334 Moscow, Russian Federation; Institute of Gene Biology, Russian Academy of Sciences, 119334 Moscow, Russian Federation; Institute of Gene Biology, Russian Academy of Sciences, 119334 Moscow, Russian Federation; Institute of Gene Biology, Russian Academy of Sciences, 119334 Moscow, Russian Federation; Department of Infectious Diseases, University Hospital of Heidelberg, Heidelberg, Germany; Department of Infectious Diseases, University Hospital of Heidelberg, Heidelberg, Germany; Department of Infectious Diseases, University Hospital of Heidelberg, Heidelberg, Germany; Department of Drug Discovery and Biomedical Sciences, College of Pharmacy, University of South Carolina, Columbia, SC 29208, USA; Department of Drug Discovery and Biomedical Sciences, College of Pharmacy, University of South Carolina, Columbia, SC 29208, USA; Department of Drug Discovery and Biomedical Sciences, College of Pharmacy, University of South Carolina, Columbia, SC 29208, USA

## Abstract

We have conducted a detailed transcriptomic, proteomic and phosphoproteomic analysis of CDK8 and its paralog CDK19, alternative enzymatic components of the kinase module associated with transcriptional Mediator complex and implicated in development and diseases. This analysis was performed using genetic modifications of CDK8 and CDK19, selective CDK8/19 small molecule kinase inhibitors and a potent CDK8/19 PROTAC degrader. CDK8/19 inhibition in cells exposed to serum or to agonists of NFκB or protein kinase C (PKC) reduced the induction of signal-responsive genes, indicating a pleiotropic role of Mediator kinases in signal-induced transcriptional reprogramming. CDK8/19 inhibition under basal conditions initially downregulated a small group of genes, most of which were inducible by serum or PKC stimulation. Prolonged CDK8/19 inhibition or mutagenesis upregulated a larger gene set, along with a post-transcriptional increase in the proteins comprising the core Mediator complex and its kinase module. Regulation of both RNA and protein expression required CDK8/19 kinase activities but both enzymes protected their binding partner cyclin C from proteolytic degradation in a kinase-independent manner. Analysis of isogenic cell populations expressing CDK8, CDK19 or their kinase-inactive mutants revealed that CDK8 and CDK19 have the same qualitative effects on protein phosphorylation and gene expression at the RNA and protein levels, whereas differential effects of CDK8 versus CDK19 knockouts were attributable to quantitative differences in their expression and activity rather than different functions.

## Graphical Abstract

**Graphical Abstract:**
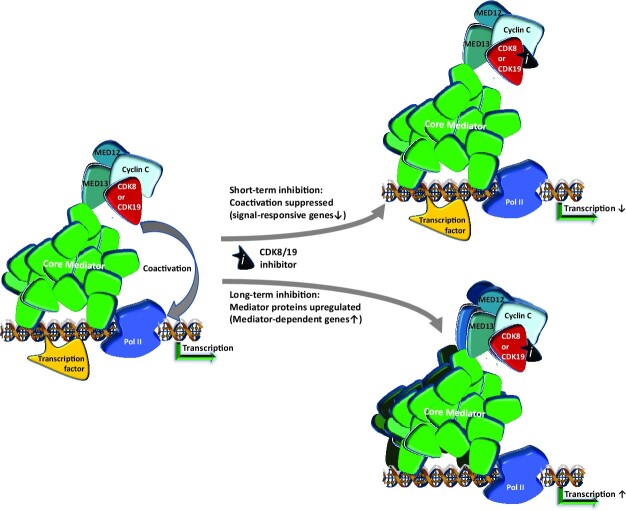

## INTRODUCTION

Transcription-regulating kinase CDK8 and its closely related paralog CDK19 are alternative enzymatic components of the kinase module associated with the transcriptional Mediator complex. The Mediator kinase module includes CDK8 or CDK19, together with their binding partner cyclin C (CCNC) and proteins MED12 and MED13 (1,2). CDK8/19 Mediator kinases co-activate several transcription factors, including β-catenin/TCF/LEF (3), SMADs (4,5), HIF1α (6) and factors regulating the glycolysis pathway (7), STATs (8), estrogen receptor (ER) (9) and NFκB (10). In some cases, Mediator kinases phosphorylate transcription factors, such as E2F1 (11), SMADs (4) and STATs (8,12), modulating their transcriptional activities and protein stability. Mediator kinases were also implicated in negative regulation of super-enhancer-driven transcription, and their inhibition in leukemia cells further increased the expression of super-enhancer-associated genes (13). Both CDK8 (14,15) and CDK19 (16) were reported to exert some of their phenotypic effects in a kinase-independent manner but no specific mechanisms of kinase-independent CDK8/19 activity have been elucidated.

In several cases, Mediator kinase activity was found to affect (possibly indirectly) the phosphorylation of the C-terminal domain (CTD) of RNA polymerase II (Pol II). The Pol II CTD phosphorylation-based mechanism has been implicated in downstream potentiation of the serum response network (17), HIF1*α* (6), ER (9) and NFκB (10) by CDK8/19. Importantly, Mediator kinase inhibition suppresses CTD phosphorylation not globally but only in the context of newly activated genes, and CDK8/19 inhibitors suppress *de novo* induction of Mediator kinase co-regulated signal-stimulated genes (10). This pattern suggested that CDK8/19 regulate transcriptional reprogramming (1,10). Transcriptional reprogramming is critical for several biological and pathological processes that are suppressed by CDK8/19 inhibition, including embryonic development (2,18), cancer metastasis (19) and drug resistance (20,21).

Since Mediator kinases have been implicated in many tumor-promoting activities, the development of CDK8/19 inhibitors has become a burgeoning area in cancer therapeutics (22). CDK8/19 inhibitors were also found to have therapeutic activities beyond oncology, such as inhibiting viral replication (23) and ameliorating autoimmune responses (24,25). Almost all the reported Mediator kinase inhibitors have similar potency against CDK8 and CDK19, and it is unknown if selective inhibition of one of the paralogs would be advantageous or detrimental for therapeutic purposes. CDK8 and CDK19 differ in their relative expression in different tissues, CDK19 being tissue-specific and CDK8 relatively ubiquitous (26). Transcriptomic effects of CDK8 and CDK19 knockdown in HeLa cells were reported to be similar although some genes appeared to be preferentially affected by either CDK8 or CDK19 (26). CDK8 and CDK19 were found to cooperate with each other in supporting leukemia cell growth (13), stimulating NFκB-induced transcription (10,27) and Dengue virus replication (23). In contrast, another study (12) concluded that CDK8 and CDK19 have mechanistically distinct functions in IFNγ-treated cells, wherein CDK8 kinase mediates IFNγ-induced transcription and STAT1 phosphorylation at S727 but CDK19 has no effect on STAT1 S727 phosphorylation and affects transcription in a kinase-independent manner. These conclusions were based principally on the findings that CDK8 knockout or inactivation mimicked the effects of a Mediator kinase inhibitor, whereas CDK19 knockdown in mouse embryo fibroblasts (MEF) had no significant effect on STAT1 S727 phosphorylation or on the expression of IFNγ-inducible genes (12).

We have now investigated the transcriptomic, proteomic and phosphoproteomic effects of CDK8 and CDK19 in human cells, using isogenic cell populations expressing either wild type (WT) or kinase-inactive versions of CDK8 or CDK19, highly selective Mediator kinase inhibitors and a CDK8/19-degrading PROteolysis TArgeting Chimera (PROTAC). Our results demonstrate that CDK8 and CDK19 have the same qualitative effects on gene expression and protein phosphorylation, including STAT1 S727 phosphorylation. The differences between the phenotypic effects of the knockout of CDK8 or CDK19 alone could be explained by quantitative differences in the expression and activity of the corresponding proteins. Transcriptomic effects of CDK8/19 were kinase-dependent, but CDK8 and CDK19 protected their binding partner CCNC from proteolytic degradation in a kinase-independent manner. Analysis of the effects of Mediator kinase inhibition on gene expression affected by different signals revealed that CDK8/19 maximize the expression of the most strongly signal-inducible genes. Mediator kinase inhibition in unstimulated cells initially leads to downregulation of a small number of genes, most of which were signal-inducible. Prolonged Mediator kinase inhibition or genetic inactivation led to upregulation of a larger gene set and a post-transcriptional increase of the protein components of both the core Mediator complex and its kinase module, suggesting a previously unknown mechanism for negative regulation of gene expression by Mediator kinases.

## MATERIALS AND METHODS

### Materials and reagents

All the key resources used in this study (reagents, cell lines, antibodies, vectors, kits, software) are listed in Supplementary Table S1.

### Cell culture

Cell lines HEK 293 (and its derivatives), HAP1, HCT116, HeLa and HT1080 were maintained in DMEM-high glucose media (Thermo-Fisher Scientific) supplemented with 10% fetal bovine serum (FBS) (Atlanta Biologics), 1% penicillin-streptomycin and 2 mM l-glutamine. Cell line MV4-11 was cultured in RPMI-1640 supplemented with 10% FBS, 1% penicillin-streptomycin and 2 mM l-glutamine. Cell line 22Rv1 and its derivatives were cultured in RPMI-1640 supplemented with 10% FBS, 1% penicillin–streptomycin, 2 mM l-glutamine, 1 mM sodium pyruvate, 0.15% sodium bicarbonate, 10 mM HEPES and 25 mM d-glucose. All cell lines were confirmed mycoplasma-free (MycoAlert PLUS mycoplasma detection kit, Lonza). For RNA analysis (RNA-Seq or qPCR), cells were seeded in 12-well plates at appropriate numbers (5 × 10^4^–3 × 10^5^ cells per well) to allow cells to grow to ∼90% confluence at the endpoint. For inhibitor treatment, cells were seeded 24 h before being treated with vehicle control (0.1% DMSO) or indicated chemicals at the stated concentrations and time periods (up to 3 days). For long-term treatment (15 days), cells were cultured in presence of vehicle or inhibitors and passaged every 3 days with replacement of fresh vehicle or inhibitors. For serum stimulation, cells were serum-starved for 48 h in serum-free media before adding FBS to final serum concentration of 10%. For signal stimulation, cells were seeded in regular culture media for 24 h and then treated with different stimulants (10 ng/ml TNF, 5–20 ng/ml IFNγ or 30 nM PMA). CDK8/19 inhibitor (Senexin B, 1 μM) was added 1 h before signal stimulation and maintained till the end of experiment.

### CDK8/19 expression and knockout vectors

Lentiviral constructs for wild-type and mutant CDK8 (pHIV-dTomato-CDK8 and pHIV-dTomato-CDK8M) were constructed by cloning full-length cDNA of human CDK8 and kinase-inactive mutant CDK8-D173A (kindly provided by Dr H. Kiaris, University of South Carolina (USC)) into lentiviral vector pHIV-dTomato (Addgene #21374). Lentiviral construct for wild-type CDK19 (pHIV-dTomato-CDK19) was generated by cloning full-length human CDK19 cDNA from F-CDK8L plasmid (Addgene #24762) into pHIV-dTomato. Construct expressing kinase-inactive mutant CDK19-D173A (pHIV-dTomato-CDK19M) was generated by cloning CDK19 cDNA (synthesized by GenScript) carrying GAC to GCC mutation in codon 173, into pHIV-dTomato. The second lentiviral vector pHIV-Luc-BlastR was constructed by replacing the ZsGreen-coding sequences with Blasticidin-resistance gene (BlastR) in the lentiviral vector pHIV-Luc-ZsGreen (Addgene #39196). pHIV-Luc-BlastR based constructs for expressing CDK8/CDK8M/CDK19/CDK19M were constructed by recloning the corresponding full-length cDNA from the corresponding pHIV-dTomato-based vectors. Gene-specific CRISPR knockout lentiviral constructs (lentiCRISPR-Puro-sgCDK8, lentiCRISPR-Blast-sgCDK19 and lentiCRISPR-sgCDK19-2) were generated by cloning annealed double-stranded oligos with gene-specific sgRNA sequences (listed in Supplementary Table S2) into BsmBI site of lentiviral vector lentiCRISPR v2 (Addgene #52961) or lentiCRISPR v2-Blast (Addgene #83480).

### Generation of CDK8/CDK19 knockout and re-expression derivatives

Generation of derivatives of 293 cells with knockout of CDK8 alone (8KO), CDK19 alone (19KO) and both CDK8 and CDK19 (dKO) was described before (27). CDK8/19 knockout derivatives of HCT116, HeLa and 22Rv1 cells were generated using lentiCRISPR-Puro-sgCDK8 and lentiCRISPR-Blast-sgCDK19-based lentiviral transductions at the Functional Genomics Core (FGC) of the USC Center for Targeted Therapeutics (CTT). The clones (HeLa-8KO, HeLa-19KO, HCT116-8KO, HCT116-19KO and 22Rv1-8KO) with complete knockout of the target protein and unaltered expression of the other paralog were selected. 22Rv1-19KO and 22Rv1-dKO derivatives were selected from cells transduced by both sgCDK8 and sgCDK19 viruses by the same procedure. HAP1 parental and 8KO cells were purchased from Horizon. HAP1-19KO and HAP1-dKO cells were generated by lentiviral transduction of parental HAP1 or HAP1-8KO cells with lentiCRISPR-sgCDK19-2. CDK8/19 knockout clones were confirmed by genomic DNA sequencing and immunoblotting analysis. For Mediator kinase re-expression, 293-dKO and HCT116-8KO cells were transduced with pHIV-dTomato-CDK8/CDK8M/CDK19/CDK19M lentiviral constructs or pHIV-dTomato vector. The dTomato-positive cells were isolated by two rounds of sorting using FACS Aria III (BD Biosciences) at the Microscopy and Flow Cytometry Core of the CTT to achieve > 95% positive cell populations named 293-dKO-V, 293-dKO-8, 293-dKO-8M, 293-dKO-19, 293-dKO-19M, HCT116-8KO-CDK8, HCT116-8KO-CDK8M, HCT116-8KO-CDK19 and HCT116-8KO-CDK19M. Parental 293 and 293-dKO cells were transduced with pHIV-Luc-BlastR-based lentiviral constructs (pHIV-Luc/CDK8/CDK8M/ CDK19/CDK19M-BlastR) and selected with Blasticidin (5 μg/ml) for 2 weeks, to obtain Blasticidin-resistant cell populations named 293-WT-V, 293-WT-8, 293-WT-8M, 293-WT-19, 293-WT-19M, 293-dKO-V’, 293-dKO-8′, 293-dKO-8M’, 293-dKO-19′ and 293-dKO-19M’.

### Generation of antibodies against CDK19, MED12 and MED13

Coding sequences for 379–473 aa of CDK19 (Q9BWU1), 607–849 aa of MED13 (Q9UHV7) and 1722–2013 aa of MED12 (Q93074) were cloned into pGEX5.1 and pET28 expression vectors. Recombinant GST-tagged CDK19 and His-tagged MED12 or MED13 epitope proteins were purified and used for immunization of rabbits and goats to generate target-specific polyclonal antibodies.

### Whole cell extracts and immunoblotting analysis

Cells grown and treated in P100 plates were washed with cold PBS twice and then lysed in RIPA lysis buffer (50 mM Tris–HCl, pH 8.0; 150 mM NaCl; 5 mM EDTA; 0.5 mM EGTA; 1% Igepal CA-630 (NP-40); 0.1% SDS; 0.5% Na deoxycholate) or IP lysis buffer (10 mM Tris–HCl, pH 7.4; 150 mM NaCl; 1 mM EDTA; 1 mM EGTA; 1% Igepal CA-630) supplemented with 1x protease/phosphatase inhibitor cocktail (Thermo-Fisher Scientific #78438), 2 mM Na_3_VO_4_ and 10 mM NaF. Ice-cold lysate was briefly sonicated to solubilize chromatin proteins before centrifugation. Protein concentrations were determined using the DC protein assay (Bio-Rad). Lysate samples with the same amount of total protein (40–50 μg) were mixed with 4× Laemmli Sample Buffer (Bio-Rad, with 2-mercaptoethanol) and run on 4–12% Express-Plus PAGE gels in Tris–MOPS (SDS) running buffer (GenScript). Proteins were transferred to PVDF membranes, blocked with 5% non-fat milk and incubated with primary and then secondary antibodies (detailed information is in Supplementary Table S1). Bands were visualized with Western Lighting Plus ECL detection reagent (Perkin Elmer, Waltham, MA, USA) using ChemiDoc Touch™ (Bio-Rad). Image processing and densitometry analysis were performed using ImageLab software (Bio-Rad).

### Stoichiometry determination for CDK8/CDK19 proteins

Cells were grown to 90% confluence in P150 plates, trypsinized, washed by PBS twice, lysed in RIPA buffer and whole cell extracts were prepared as above. For quantification of CDK8 to CDK19 protein ratio in 293 cells, 293 cell extracts or recombinant GST-CDK8 and GST-CDK19 proteins serially diluted with 293-dKO whole cell extracts (to equalize total protein amounts per lane) were analyzed by immunoblotting with anti-CDK8, anti-CDK19 and anti-GST antibodies. Band signal intensities were acquired using ImageLab software (Bio-Rad) and further analyzed in Microsoft Excel to evaluate the linear relationship between band signals and serial dilutions and quantify relative CDK8 and CDK19 protein levels. Only the standard points that gave a good linear regression were used to build calibration curves and only the lanes/dilutions of those cell extracts whose band signals fall in the linear range were picked for quantitation. GST band signals were used to normalize the levels of the two GST recombinant proteins. This normalization was used to adjust the ratio of CDK8 to CDK19 calculated from the quotient of relative CDK8 and CDK19 protein levels determined by mapping CDK8/CDK19 band signals to standard curves generated with GST recombinant proteins. For quantification of CDK8/CDK19 ratios in other cell lines, serially diluted cell extracts were run in parallel with 293 whole cell extract as an internal standard for quantification. Relative abundance of CDK8 and CDK19 proteins between the tested cell lines and 293 was determined by densitometry and used to calculate CDK8/CDK19 ratios in each cell line.

### RNA-seq analysis

Cells seeded in 12-well plates and treated as described previously and in figure legends were lysed for RNA extraction using RNeasy Mini Kit (Qiagen). RNA-Seq library preparation, NGS, post-processing of the raw data and data analysis were performed by Functional Genomics Core (FGC) of the CTT. RNA-Seq libraries were prepared in conjunction with poly(A)-enrichment using either TruSeq Stranded mRNA prep kit (RS-122-2101/RS-122-2102) or NEBNext Ultra II Directional RNA Library Prep Kit (#E7760). NGS was performed on Illumina NextSeq 500 (at FGC) or HiSeq 3000/4000 (at Genewiz, Inc., South Plainfield, NJ) or Illumina NovaSeq (at MedGenome, Inc., Foster City, CA) platforms for paired end sequencing. Reads were mapped to the Human GRChg38 reference genome using STAR v2.4 (28). Samtools (v1.5) was used to convert aligned SAM files to BAM files, and reads were counted using the featureCounts function of the Subreads package (29) with Gencode.v25.basic.annotation.gtf annotation file. Only reads that were mapped uniquely to the genome were used for gene expression analysis. Differential expression (DE) analysis was performed in R using the DESeq2 (30) pipeline, where the normalized counts data were fit to a negative binomial distribution model using a generalized linear model (GLM) framework and the Benjamini-Hochberg procedure was used to control the false discovery rate (FDR) for multiple testing. The logFC and FDR values calculated from DESeq2 pipelines were utilized to select differentially expressed genes (DEGs). To select high-confidence DEGs from multiple RNA-Seq experiments, the following criteria were applied: (i) FDR < 0.05 in all experiments; (ii) average log_2_FC from multiple experiments > log_2_(1.5). Normalized RNA expression levels of CDK8 and CDK19 in different cancer cell lines were retrieved from Cancer Cell Line Encyclopedia (CCLE) database (CCLE_RNAseq_genes_rpkm_20180929.gct.gz). RNA-Seq raw data of MEF treated with IFNγ and CA (12) were downloaded as SRR files from NCBI-SRA website and converted into Fastq files using SRA Toolkit. Reads were mapped to the Mouse GRCm38.88 reference genome and processed to gene counts with Gencode.vM15 annotation file for DE analysis. All raw RNA-Seq data have been uploaded to GEO (see data availability section) and detailed information about individual RNA-Seq samples (sample title and description, GEO accession number) is listed in Supplementary Table S3.

#### Quantitative RT-PCR (qPCR)

Cells were seeded in 12-well plates at the density required to approach confluence at the end of experiment and treated as indicated in figure legends before being lysed for RNA extraction using RNeasy Mini Kit (Qiagen). One microgram of total RNA was used to generate cDNA using iScript cDNA synthesis kit (Bio-Rad). Target gene expression was quantified using iTaq Universal SYBR green super mix in CFX384 Real time system (Bio-Rad). Primers used for RT-PCR are listed in Supplementary Table S2. RT-PCR data files were processed using Bio-Rad CFX Manager software to retrieve Ct numbers of qPCR reactions. Relative RNA expression of specific genes was calculated by the formula: Relative Expression = 2^(Ct_reference_ – Ct_gene_), where RPL13A, HPRT1 or GAPDH were used as reference genes.

#### Proteomics and phosphoproteomics analysis

Tandem Mass Tag (TMT) based proteomic and phosphoproteomic analysis of a total of 30 samples was performed in three TMT-11plex batches. The first batch comprised dKO-CDK8 and dKO-CDK8M (five biological replicates of each), the second dKO-CDK19 versus dKO-CDK19M (5 + 5 replicates), and the third parental 293 cells treated with DMSO (Ctrl), 1 μM Senexin B (3 h) or 1 μM Senexin B (72 hrs) (4 + 3 + 4 replicates, correspondingly). For each replicate, cells were grown in a P150 plate to ∼90% confluence before being collected for analysis. At the endpoint, culture media were removed and cells were rinsed with ice-cold PBS three times and scraped down in 5 ml PBS with protease/phosphatase inhibitor cocktail. Cells were pelleted by centrifugation at 200 g × 5 min at 4°C, snap-frozen after removal of supernatant and stored at –80°C before the proteomics/phosphoproteomics analysis at the Proteomics Core Laboratory of the University of Arkansas for Medical Sciences (UAMS). Cells were lysed in RIPA buffer (Thermo-Fisher Scientific PI89901) and 200 μg total protein lysates were reduced, alkylated and purified by chloroform/methanol extraction prior to digestion with sequencing grade trypsin and LysC (Promega). The resulting peptides were labeled using a TMT 11-plex isobaric label reagent set (Thermo-Fisher Scientific) in three multiplex batches with a pooled reference sample in each batch, then enriched using High-Select TiO2 and Fe-NTA phosphopeptide enrichment kits (Thermo-Fisher Scientific) following the manufacturer's instructions. Both enriched and unenriched labeled peptides were separated into 46 fractions on a 100 × 1.0 mm Acquity BEH C18 column (Waters) using an UltiMate 3000 UHPLC system (Thermo-Fisher Scientific) with a 50 min gradient from 99:1 to 60:40 buffer A:B ratio under basic pH conditions, then consolidated into 18 super-fractions. Each super-fraction was further separated by reverse phase XSelect CSH C18 2.5 um resin (Waters) on an in-line 150 × 0.075 mm column using an UltiMate 3000 RSLCnano system (Thermo-Fisher Scientific). Peptides were eluted using a 75 min gradient from 98:2 to 60:40 buffer A:B ratio. Eluted peptides were ionized by electrospray (2.2 kV) followed by mass spectrometric analysis on an Orbitrap Eclipse Tribrid mass spectrometer (Thermo-Fisher Scientific) using multi-notch MS3 parameters with real-time search enabled. MS data were acquired using the FTMS analyzer in top-speed profile mode at a resolution of 120 000 over a range of 375–1500 *m*/*z*. Following CID activation with normalized collision energy of 31.0, MS/MS data were acquired using the ion trap analyzer in centroid mode and normal mass range. Using synchronous precursor selection, up to 10 MS/MS precursors were selected for HCD activation with normalized collision energy of 55.0, followed by acquisition of MS3 reporter ion data using the FTMS analyzer in profile mode at a resolution of 50 000 over a range of 100–500 *m*/*z*. Proteins and phosphosites were identified and reporter ions quantified by searching the UniprotKB H. sapiens database (July 2020) using MaxQuant (version 1.6.17.0; Max Planck Institute) with a parent ion tolerance of 3 ppm, a fragment ion tolerance of 0.5 Da, a reporter ion tolerance of 0.001 Da, trypsin enzyme with 2 missed cleavages, variable modifications including oxidation on M, Acetyl on Protein N-term, and phosphorylation on STY, and fixed modification of carbamidomethyl on C-term. Protein and peptide identifications were accepted if established with <1.0% false discovery. TMT MS3 reporter ion intensity values were analyzed for changes in total protein using the unenriched lysate sample. Phospho (STY) modifications were identified using the samples enriched for phosphorylated peptides. The enriched and unenriched samples were multiplexed using two TMT11-plex batches, one for the enriched and one for the unenriched samples. Following data acquisition and database search, the results were normalized using cyclic loess normalization for both the protein and the phosphopeptide data sets (31). The normalized protein and phosphorylated peptide data were analyzed for differential abundance using the limma package by applying ‘lmFit’ and ‘eBayes’ functions. A similar approach was used for differential analysis of the phosphopeptides. The phosphosites were filtered to retain only peptides with a localization probability >75% and log_2_ cyclic loess transformed. Limma is also used for differential analysis of single phosphosite peptides. The *P*-values were adjusted for multiple test correction using the false discovery rate (FDR). The raw and processed proteomics and phosphoproteomics data have been uploaded to MassIVE database (see data availability section).

#### Statistical analysis

WB experiments were performed at least in duplicates. Means of densitometry signals from WB duplicate images are presented in the bar diagrams. RNA-Seq experiments were carried out in biological replicates (*n* ≥ 3) for each treatment condition. Procedures for RNA-Seq and proteomics data analysis are described in the above sections. The significance of the overlap detected in Venn diagrams was assessed by a hypergeometric test. Slope and Pearson correlation coefficients were calculated by linear regression and correlation analysis using GraphPad Prism 9 software. qPCR analysis was performed in biological triplicates and data were presented as mean ± standard error of the mean (SEM). Statistical significance was tested using ordinary two-way ANOVA and Tukey's multiple comparisons test with GraphPad Prism 9 software.

## RESULTS

### Experimental strategy

**Figure 1.**
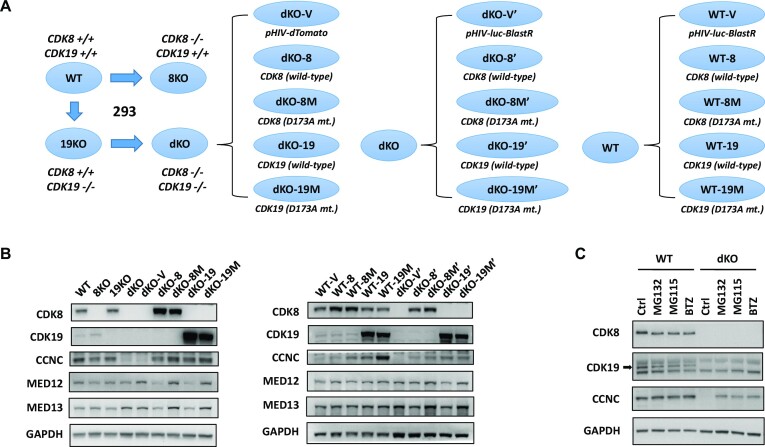
Effects of CDK8 and CDK19 expression and kinase activity on protein expression of Mediator kinase module components. (**A**) Scheme of generating CDK8/19 single- and double-knockout and reconstitution derivatives in 293 cells. (**B**) Immunoblotting analysis of CDK8/19 derivatives in 293 cells for CDK8, CDK19, CCNC, MED12, MED13 and GADPH (normalization standard). (**C**) Parental (WT) and dKO cells were treated with 0.1% DMSO (Ctrl) or proteasome inhibitors (5 μM MG132, 5 μM MG115 or 5 μM Bortezomib (BTZ)) for 18 h and analyzed by immunoblotting for CDK8, CDK19, CCNC and GAPDH.

As the primary cellular model to analyze the transcriptomic and proteomic effects of CDK8 and CDK19, we have used 293 human embryonic kidney cells, the principal cell line used in our earlier study that elucidated the role of CDK8/19 in NFκB signaling (10). We have previously generated 293 derivatives with the CRISPR-mediated knockout of CDK8 alone (8KO), CDK19 alone (19KO) and both CDK8 and CDK19 (double knockout, dKO) (27). However, we avoided drawing conclusions from comparisons between these knockout clones and the parental 293 cells for the following reasons: (i) comparison of parental cells to individual subclones reveals numerous differences, especially at the transcriptomic level, due to clonal variability and (ii) sgRNA knockout (or siRNA knockdown) causes transcriptomic changes that are not necessarily mediated by the target. Instead, our principal analysis was based on comparing isogenic mass populations of dKO cells reconstituted with wild-type or kinase-inactive versions of CDK8 or CDK19, with further validation of the conclusions using highly selective small-molecule inhibitors and a novel PROTAC degrader of CDK8/19. Cell line derivatization strategy is diagrammed in Figure 1A. We have transduced dKO cells with a lentiviral vector (pHIV-dTomato) expressing either wild-type CDK8 or CDK19 or their kinase-inactive D173A mutants (16,32), obtaining mass populations named dKO-8, dKO-8M, dKO-19 and dKO-19M. dKO cells transduced with an insert-free vector (dKO-V) were used as a control. To assure reproducibility, a second set of dKO derivatives was generated using another lentiviral vector (pHIV-Luc-BlastR) (derivatives were named dKO-8′, dKO-8M’, dKO-19′ and dKO-19M’); dKO cells transduced with the luciferase-expressing vector (dKO-V’) were used as a control. Since Mediator kinase expression levels in the dKO derivatives were expected to be higher than in the original 293 cells, and because protein overexpression could cause artificial changes, we have introduced an additional layer of controls to account for the effects of the overexpression, by generating and analyzing derivatives of the parental 293 cells that were made to overexpress CDK8, CDK19 or their kinase-inactive mutants from pHIV-Luc-BlastR (these cell populations were named WT-V, WT-8, WT-8M, WT-19 and WT-19M) (Figure 1A). This set of 293 cell derivatives was used to derive our initial conclusions, which were then tested using selective small-molecule kinase inhibitors and a targeted degrader of CDK8/19.

### Effects of CDK8 and CDK19 on CCNC degradation

Immunoblotting analysis in Figure 1B verifies the knockout and reconstitution of CDK8 and CDK19 in 293 cells and asks if CDK8/19 expression affects the other components of the Mediator kinase module: CCNC, MED12 and MED13. Remarkably, the levels of CCNC, the necessary binding partner of CDK8 and CDK19, were drastically decreased in dKO, whereas reconstitution of either WT or kinase-inactive mutant CDK8 or CDK19 in these cells restored CCNC levels (Figure 1B). To determine if CCNC stabilization by CDK8 and CDK19 was due to protection from proteasomal degradation, we have tested the effects of three proteasome inhibitors: MG132, MG115 and bortezomib, on CCNC protein levels in the parental and dKO cells. All three inhibitors had no significant effect on CCNC in the parental cells but greatly increased its levels in dKO (Figure 1C), indicating the role of proteasomal degradation in the regulation of CCNC levels by Mediator kinases. Notably, expression of kinase-inactive CDK8 or CDK19 mutants in dKO cells not only restored CCNC expression but did so to a greater extent than their WT counterparts (Figure 1B). Furthermore, overexpression of kinase-inactive (but not WT) CDK8 or CDK19 further increased CCNC in parental cells (Figure 1B). These results are in agreement with an earlier study that found CCNC to be stabilized by both kinase-active and inactive CDK8 (33). In contrast to CCNC, MED12 and MED13 proteins were not downregulated but in fact slightly increased in dKO or dKO-V cells, and their levels were decreased by the expression of the WT but not kinase-inactive CDK8 or CDK19 in dKO (Figure 1B). These results suggested that CDK8 and CDK19 protect CCNC (but not MED12 or MED13) from proteolytic degradation in a kinase-independent manner, whereas their kinase activity may have a negative effect on all three of the other components of the Mediator-associated CDK module. As described below, proteomic analysis demonstrated a broad negative effect of Mediator kinase activity on the protein levels of not only the CDK module but also the core Mediator components.

### CDK8 and CDK19 have similar, kinase-dependent effects on basal gene expression

**Figure 2.**
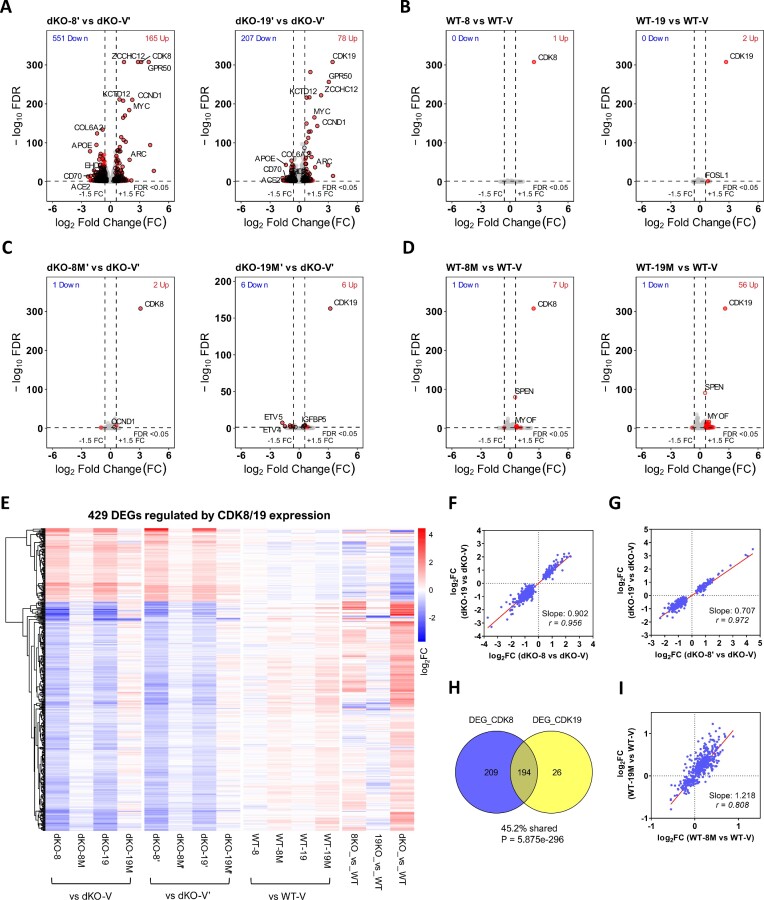
RNA-Seq analysis of the effects of CDK8 and CDK19 knockout and re-expression on gene expression. (**A**) Volcano plots of comparisons of gene expression between 293 dKO cells reconstituted with WT CDK8 or CDK19 relative to the corresponding vector-transduced dKO controls. Red dots: DEGs passing the selection criteria (FC > 1.5; FDR < 0.05). Black circles: high-confidence DEGs that pass the selection criteria (average FC > 1.5; FDR < 0.05 in both series of reconstitution derivatives, see Supplementary Figure S1A). (**B**) Volcano plots of comparisons of gene expression between parental 293 (WT) cells overexpressing wild-type CDK8 or CDK19 relative to the corresponding vector-transduced WT controls. (**C**, **D**) Volcano plots of comparisons of gene expression between 293 dKO (C) or WT cells (D) expressing kinase-inactive CDK8 (8M) or CDK19 (19M) mutants relative to the corresponding vector-transduced controls. (**E**) Heatmap of 429 high-confidence DEGs regulated by CDK8/19 reconstitution in dKO cells in 293 derivatives (hierarchical clustering). (**F, G**) Comparison of the effects of CDK8 versus CDK19 reconstitution in dKO cells on the high-confidence CDK8/19-regulated DEGs in two different series of reconstitution derivatives. (**H**) Overlap of DEGs affected by CDK8 or CDK19 reconstitution in dKO cells; *P*-value determined by hypergeometric test. (**I**) Comparison of the effects of kinase-inactive CDK8 versus kinase-inactive CDK19 expression in parental cells on the high-confidence CDK8/19-regulated DEGs. Slope and Pearson correlation coefficients (r) were calculated by linear regression and correlation analysis.

RNA-Seq analysis was used to characterize the transcriptomic effects of wild-type or kinase-inactive CDK8 or CDK19 expression in dKO and WT 293 cells. The strategy for the selection of Differentially Expressed Genes (DEG) is diagrammed in Supplementary Figure S1A; fold-change (FC) >1.5 and False Discovery Rate (FDR) <0.05 were used as the cutoff criteria for DEG selection. Figure 2A shows volcano plots of the effects of the expression of WT CDK8 or CDK19 in dKO cells, with black circles marking the high-confidence DEGs that were shared in two independent sets of derivatives. Four hundred and three high-confidence DEGs were regulated by WT CDK8 and 220 DEGs by WT CDK19; these overlapping sets comprise a total of 429 high-confidence DEGs regulated by WT CDK8 or CDK19 (Supplementary Table S4). In contrast to their effects in dKO cells, overexpression of WT CDK8 or CDK19 in parental cells had no significant effects on gene expression (Figure 2B), validating the use of ectopically expressed Mediator kinases in our experimental strategy.

Unlike WT CDK8 and CDK19, very few genes were regulated by kinase-inactive Mediator kinase mutants in dKO cells, with only 11 high-confidence genes weakly affected by the CDK19 mutant and 2 genes by the CDK8 mutant (Figure 2C), indicating that regulation of gene expression by CDK8 and CDK19 is largely kinase-dependent. This conclusion was confirmed by PROTAC analysis (see below). On the other hand, kinase-inactive CDK8 and CDK19 mutants showed detectable effects on gene expression in parental cells (Figure 2D), which were qualitatively similar to the effect of dKO (Figure 2E), indicating that both mutant proteins exert a moderate dominant negative effect on the endogenous Mediator kinases.

Transcriptomic effects of CDK8 and CDK19 are compared in Figure 2E–I. Figure 2E shows the heatmap with hierarchical clustering of 429 CDK8/19-regulated high-confidence DEGs in all the derivatives. Reconstitution of CDK8 or CDK19 in dKO cells had the same qualitative effects on all the DEGs but the effects of CDK19 were quantitatively weaker (Figure 2F, G). Notably, this quantitative difference is more pronounced in the second set of reconstituted cell lines (Figure 2G), which expressed CDK19 at a lower level than the first set (Figure 1B). This difference is also apparent from the Venn diagram in Figure 2H comparing DEGs selected from CDK8 or CDK19 expression in dKO cells. While the overlap was highly significant and almost all the CDK19-regulated DEGs were also CDK8-regulated, one half of CDK8-regulated DEGs failed to pass the cutoff criteria for the effect of CDK19, despite qualitative coregulation (Figure 2F, G). Furthermore, the dominant negative effects of mutant CDK8 or CDK19 expression in parental cells were very similar but in this case the effect of mutant CDK19 was stronger (Figure 2I). Hence, CDK8 and CDK19 have qualitatively the same but quantitatively different transcriptomic effects, both as WT kinases and as dominant negative mutants.

The effects of the knockout of both CDK8 and CDK19 (dKO) on CDK8/19-regulated DEGs were inverse to the effects of CDK8 or CDK19 expression (Figure 2E), indicating that both CDK8 and CDK19 reversed the transcriptomic effects of dKO. The effects of CDK8 knockout (8KO) on CDK8/19-regulated DEGs were weaker than but similar to the effects of dKO (Figure 2E), but the effect of CDK19 knockout (19KO) was very weak and did not show a similar pattern (Figure 2E). The reasons for the difference between the CDK8 and CDK19 knockouts will be discussed below.

### Downregulation of gene expression is an early response and upregulation is a late response to CDK8/19 inhibition

**Figure 3.**
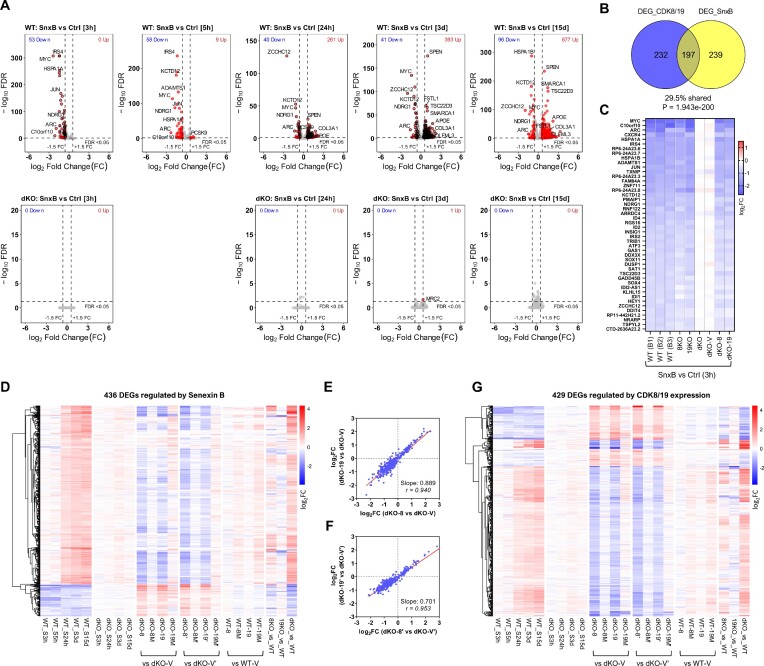
RNA-Seq analysis of the effects of CDK8/19 inhibitor treatment on gene expression. (**A**) Volcano plots of the effects of treatment with 1 μM Senexin B on gene expression in the parental (WT) cells (above) and their dKO derivative (below) for the indicated periods of time. Red dots: DEGs passing the selection criteria (FC > 1.5; FDR < 0.05). Black circles: high-confidence DEGs that pass the selection criteria (average FC > 1.5; FDR < 0.05 in all independently analyzed batches, see Supplementary Figure S1C). (**B**) Overlap of DEGs affected by Senexin B or by CDK8 or CDK19 expression. (**C**) Effects of 3-h Senexin B treatment on the expression of 46 high-confidence early-response DEGs in different batches of parental cells and the indicated derivatives. (**D**) Heatmap of 436 high-confidence DEGs regulated by Senexin B (at either 3, 24 or 72 h time points) at different timepoints of Senexin B treatment and in different 293 derivatives. (**E, F**) Comparison of the effects of CDK8 versus CDK19 reconstitution in dKO cells on the 436 high-confidence Senexin B-regulated DEGs in two different series of reconstitution derivatives. Slope and Pearson correlation coefficients (*r*) were calculated by linear regression and correlation analysis. (**G**) Heatmap of 429 DEGs regulated by CDK8/19 reconstitution in dKO cells at different timepoints of Senexin B treatment.

To confirm the transcriptomic data obtained from Mediator kinase expression and mutagenesis and to elucidate the time course of the transcriptomic effects of CDK8/19 inhibition, we have used Senexin B, a highly selective CDK8/19 inhibitor (9,34) that was the first to reach clinical trials (35). Parental 293 and dKO cells were untreated or treated with Senexin B (1 μM) for different periods of time (3 hrs to 15 days). Volcano plots of RNA-Seq data (Figure 3A) show that almost no genes were affected by Senexin B in dKO cells, confirming high target selectivity of this inhibitor. We also compared the effects of 3-day treatment with Senexin B and three other chemically unrelated selective CDK8/19 inhibitors didehydrocortistatin A (dCA) (36), 15w (27,37) and BI1347 (38) in the parental and dKO cells. Like Senexin B, dCA, 15w and BI1347 changed expression of hundreds of DEGs (more upregulated than downregulated) in WT cells and had minimal or no transcriptomic effects in dKO cells, as shown by volcano plots (Supplementary Figure S2A). The heatmap (Supplementary Figure S2B) shows the effects of Senexin B, dCA, 15w and BI1347 on 396 DEGs that were regulated after 3 day Senexin B treatment in the parental cells (Supplementary Figure S2B). The correlation analysis confirmed that the effects of the inhibitors were very similar (Supplementary Figure S2C–E), indicating that all four compounds regulated gene expression through Mediator kinase.

Selection of DEGs regulated by Senexin B at different time points is diagrammed in Supplementary Figure S1C, using FC > 1.5 and FDR < 0.05 as the cutoffs. The CDK8/19 inhibitor-regulated DEG sets at 3-h, 24-h and 3-day time points were further confirmed (black-circled in the volcano plots, Figure 3A) by the concordance among three (for 3- and 24-h) or two batches (for 3-day points) of independent RNA-Seq studies conducted at different times (Supplementary Figure S1B). Forty six high-confidence DEGs (all downregulated) were affected at 3-h, 123 DEGs (100 up and 23 down) at 24-h and 396 DEGs (359 up and 37 down) at 3-day time points, yielding a combined total of 436 DEGs that are regulated by Senexin B at any of the three time points (Supplementary Table S5). This DEG set significantly overlaps with the DEGs selected on the basis of CDK8/19 expression (Figure 3B) despite the stringent cutoff criteria for DEG selection.

Changes in gene expression upon CDK8/19 inhibition can be seen in Figure 3A (volcano plots) and in the heatmaps in Figure 3C, D. At the earliest time point (3 h), Senexin B downregulated only 46 genes in all three independent studies, indicating that Mediator kinases act as positive regulators of the early-response genes (Figure 3C). The early responsive genes were regulated by the inhibitor almost identically in 8KO and 19KO single knockouts, and in dKO-8 and dKO-19 re-expressing cells, but not in dKO or dKO-V cells (Figure 3C), confirming that these genes are regulated by both CDK8 and CDK19. Figure 3D shows the complete time course of the effects of Senexin B on 436 inhibitor-regulated DEGs. While downregulation of gene expression was the primary response at 3–5 h, upregulation of gene expression became predominant at 24 h–15 days (Figure 3D). As the treatment length increased, the effects of Senexin B on DEG expression became similar to the effects of dKO (relative to parental cells) (Figure 3D, Supplementary Figure S2F–J). The effects of CDK8/19 reconstitution in dKO largely counteracted the effects of Senexin B on the inhibitor-regulated DEGs (Figure 3D), and the effects of CDK8 and CDK19 on these genes were qualitatively identical (Figure 3E, F), in agreement with their effects on genes regulated by CDK8/19 expression (Figure 2F, G). Senexin B treatment also counteracted the effects of CDK8 and CDK19 on the DEGs selected by CDK8/19 reconstitution (Figure 3G). The results of RNA-Seq were confirmed by reverse transcriptase quantitative PCR (qPCR) for the genes that were upregulated (APOE, EHD2, COL3A1) or downregulated (MYC, ZCCHC12, ARC) by Senexin B (Supplementary Figure S3A) or by a chemically unrelated CDK8/19 inhibitor SNX631 (21) (Supplementary Figure S3B).

Supplementary Figure S4A shows the effects of Senexin B treatment, WT CDK8 or CDK19 expression in dKO cells, and the knockout of CDK8, CDK19 or both Mediator kinases, on the combined set of 668 DEGs regulated by either CDK8/19 expression or Senexin B. As in the individual sets (Figure 3D, G), CDK8 or CDK19 expression in dKO cells produces the opposite effects to prolonged Senexin B treatment on the combined DEG set (except for a small number of genes affected by the inhibitor but not by Mediator kinase expression). The effects of dKO closely resemble the effects of Senexin B, whereas single knockout of CDK8 but not CDK19 partially reproduces these effects. In contrast, the frequently used approach of defining the effects of genes through their knockouts does not give such a clear pattern. Using the same cutoff criteria (FC > 1.5 and FDR < 0.05), we identified 1122 high-confidence DEGs affected in dKO relative to parental cells (Supplementary Figure S1C), 1040 DEGs affected in 8KO and 158 DEGs affected in 19KO and generated heatmaps for these DEG sets under the same conditions. Most of dKO-based DEGs are affected by Senexin B and CDK8/19 expression but some dKO-affected DEGs were neither reversed by CDK8 or CDK19 expression nor regulated by Senexin B (Supplementary Figure S4B). The number of such non-responsive genes is much greater among the 8KO-based DEGs (Supplementary Figure S4C) and especially 19KO-based DEGs (Supplementary Figure S4D). Such genes may reflect the CDK8/19-unrelated effects of sgRNA/CRISPR transduction and clonal selection, illustrating the shortcomings of the analysis based on gene knockout.

### Targeted degradation of CDK8/19 confirms kinase dependence of the transcriptomic effects and indicates compensatory transcriptomic changes in dKO

**Figure 4.**
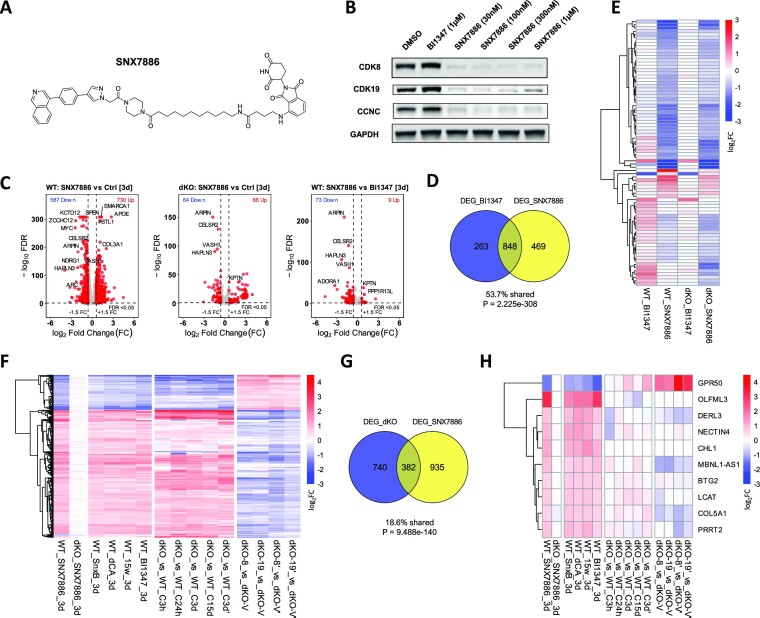
Effects of a CDK8/19-degrading PROTAC. (**A**) Chemical structure of the CDK8/19-degrading PROTAC SNX7886. (**B**) Immunoblotting analysis of CDK8, CDK19 and CCNC expression in 293 cells treated for 24 h with BI1347 or SNX7886 at the indicated concentrations. (**C**) Volcano plots of the effects of 72-hr treatment with 200 nM SNX7886 versus vehicle control (parental (WT) cells), 200 nM SNX7886 versus 200nM BI1347 (parental cells) and 200 nM SNX7886 versus vehicle control (dKO cells). (**D**) Overlap of DEGs affected by BI1347 or SNX7886 treatment. (**E**) Heatmap of 82 DEGs differentially affected by SNX7886 and BI1347 in WT cells under indicated conditions. (**F**) Heatmap of DEGs that are affected by Senexin B or CDK8/19 expression (see Supplementary Figure S4A) and regulated by SNX7886 under indicated conditions. (**G**) Overlap of DEGs affected by SNX7886 treatment or dKO. (**H**) Heatmap of the genes regulated by all CDK8/19 inhibitors or PROTAC but not by dKO under indicated conditions.

We have recently developed a potent PROTAC degrader of CDK8 and CDK19 (manuscript in preparation). This PROTAC, SNX7886 (Figure 4A) is based on the CDK8/19 inhibitor BI1347 connected to a Cereblon E3 ligase binder pomalidomide via an alkane linker. Treatment of 293 cells with SNX7886 at concentrations as low as 30 nM degrades CDK8 by up to ∼90% and CDK19 by up to ∼80%, with concurrent CCNC degradation (Figure 4B) that resembles the effects of dKO (Figure 1B).

To determine if CDK8/19 degradation would produce any transcriptomic effects distinct from those of kinase inhibition (i.e. kinase-independent effects), we have carried out RNA-Seq analysis of parental and dKO 293 cells treated for 72 hrs with 200 nM SNX7886, its cognate kinase inhibitor BI1347 or DMSO control. Volcano plots in Figure 4C show that the PROTAC affected multiple genes in the parental 293 cells and, in contrast to the kinase inhibitors, also impacted a number of genes in dKO cells (Figure 4C); the effects in dKO are most likely attributable to the PROTAC’s pomalidomide moiety. Most of the genes affected by the kinase inhibitor and the PROTAC overlapped (Figure 4D) but 82 genes were differentially affected by the PROTAC versus the kinase inhibitor; most of these genes were inhibited (Figure 4C). The heatmap in Figure 4E shows, however, that the same genes were also affected by the PROTAC in dKO (with the exception of a single gene, which, as shown below, was in fact affected by kinase inhibitors). Hence, the effects of the PROTAC that were not shared by the kinase inhibitor were not CDK8/19-mediated. Notably, none of the genes that appeared to be weakly affected by kinase-inactive CDK19 or CDK8 mutants (Figure 2C) were differentially affected by the kinase inhibitors and PROTAC. These results, together with the above-described effects of the kinase-inactive CDK8 and CDK19 mutants, confirm that the transcriptomic effects of CDK8 and CDK19 are kinase-dependent.

The heatmap in Figure 4F compares the effects of SNX7886 PROTAC and four CDK8/19 kinase inhibitors (Senexin B, dCA, 15w and BI1347), dKO (5 different studies) and CDK8 or CDK19 reconstitution in dKO cells (two different studies) on the expression of 366 DEGs affected by Senexin B or CDK8/19 reconstitution (Supplementary Figure S4A) and the PROTAC. The effects of dKO on these DEGs largely resembled the effects of the inhibitors or PROTAC and reversely correlated with the effects of CDK8 or CDK19 expression in dKO (Figure 4F). Venn diagram comparison of the effects of the PROTAC and dKO (Figure 4G) reveals the expected overlap but also more differences than in other pairwise comparisons. While dKO-specific effects are likely to stem from the clonal nature of dKO cells, there are also many genes affected by the PROTAC but not by dKO, suggesting that such genes could have undergone compensatory changes during the establishment of dKO cell line. For a closer look at such compensatory changes, we have selected a subset of DEGs that were affected (FC > 1.5, FDR < 0.05) by all 4 kinase inhibitors and the PROTAC but not affected by dKO in the same direction in any of the studies. As shown in Figure 4H, some of the genes unaffected by dKO were still affected by CDK8 or CDK19 expression in dKO, in the direction opposite to the effect of the inhibitors, whereas a few genes were unaffected in dKO cells by CDK8 or CDK19 expression suggesting that the adaptation of these cells involved a switch from CDK8/19-dependent to CDK8/19-independent regulation.

Supplementary Figure S5 illustrates the effects of all the different conditions on genes representing different CDK8/19 response patterns. Genes in Supplementary Figure S5A are upregulated by CDK8/19 expression and downregulated by Mediator kinase inhibition, with MYC and JUN affected stronger at the early than at the late timepoints of the inhibitor treatment, whereas ZCCHC12 is inhibited stronger at the later timepoints. Genes in Supplementary Figure S5B are downregulated by CDK8/19 expression and upregulated by Mediator kinase inhibition; such genes are affected stronger at the later timepoints. Genes in Supplementary Figure S5C are affected by the inhibitors or the PROTAC but not by dKO (likely compensatory changes), with GPR50 and DERL3 affected and OLFML3 unaffected by CDK8 or CDK19 expression in dKO. Finally, Supplementary Figure S5D provides examples of genes that show variable response to CDK8/19 mutants or inhibitors in different batches. Thus, RPL12P14 was differentially induced by BI1347 kinase inhibitor and SNX7886 PROTAC (Figure 4D) but Supplementary Figure S5D shows that it was still induced by BI1347 and induced even stronger by four other kinase inhibitors. Also CCND1 appears to be upregulated by kinase-inactive CDK8 and ETV5 downregulated by kinase-inactive CDK19 mutant in dKO cells but these genes were not selectively affected by the PROTAC, indicating that their regulation was not in fact kinase independent.

### Mediator kinases potentiate the induction of gene expression by different signals

Extending our previous studies on the potentiation of signal-induced transcriptional activation by CDK8/19 activity in 293 cells (10), we have investigated the effects of CDK8/19 inhibition on transcriptomic responses to a variety of transcription-altering signals in 293 cells, including serum stimulation (previously shown to be potentiated by CDK8 (17)), NFκB activation (potentiated by CDK8/19 (10)), protein kinase C (PKC) activation (not previously analyzed for Mediator kinase dependence) and IFNγ treatment (reported to be affected by Mediator kinase (8,12)). Cells were treated with the corresponding signal inducers in the presence or absence of Senexin B (1 μM), added 1 h before the signals. DEGs affected by each agent or by Senexin B were selected by the criteria FC > 1.5, FDR < 0.05; the corresponding flow charts and DEG numbers are shown in Supplementary Figure S1D.

**Figure 5.**
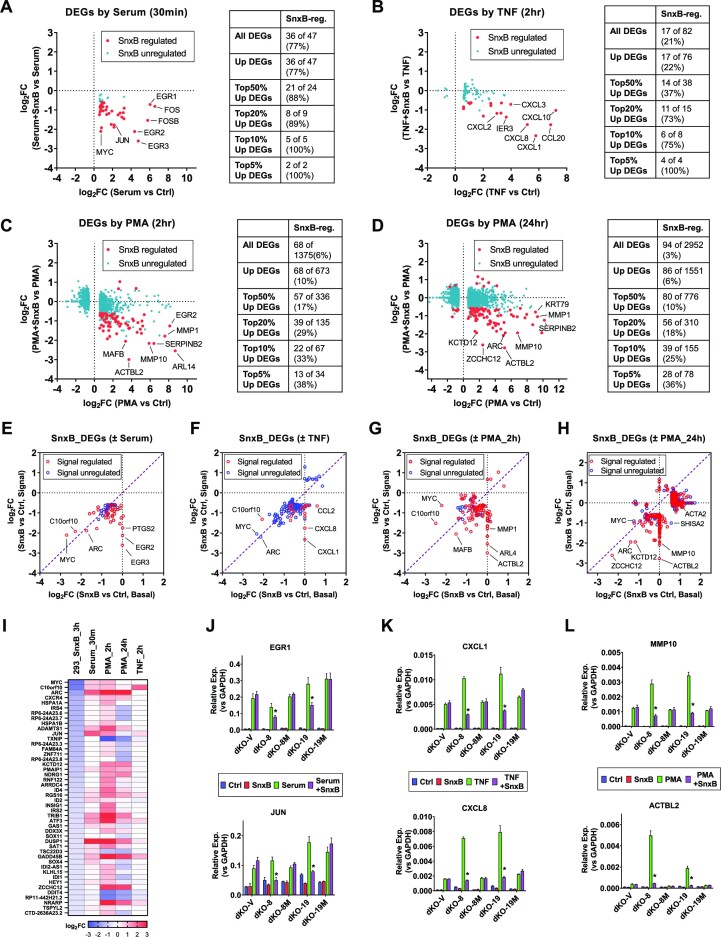
Transcriptomic analysis of the effects of Mediator kinases on signal-regulated gene expression. (A–D) RNA-Seq analysis of 293 cells treated with the indicated signals in the presence or in the absence of 1 μM Senexin B (SnxB), added 1 h before signal stimulation and maintained till the end of experiment. (**A**) Cells were serum starved for 48 h and then treated with serum (FBS added to 10% final concentration) for 30 min. (B–D) Cells were treated with TNF (10 ng/ml) for 2 h (**B**) or PMA (30 nM) for 2 h (**C**) or 24 h (**D**). The dot plots show the effects of Senexin B treatment on the signal-affected DEGs (FC > 1.5; FDR < 0.05). Red dots: Senexin B-affected DEGs. Blue dots: Senexin B-unaffected DEGs. The tables on the right show the number and percentage of signal-regulated DEGs affected by Senexin B treatment. (**E–H**) Comparison of effects of Senexin B on the expression of genes regulated by Senexin B either under basal conditions or upon signal stimulation. Red circles: signal-regulated genes. Blue circles: genes that are not regulated by signals. (**I**) Effects of different signals on the expression of 46 DEGs regulated by Senexin B at 3 h time point under basal conditions. (**J–L**) qPCR analysis of mRNA expression of the indicated genes in dKO derivatives with or without signal or Senexin B: serum stimulation (**J**), TNF (**K**), PMA (24 h) (**L**). Data are presented as mean ± SEM (*n* = 3). Asterisks: *P* < 0.01 (two-way ANOVA, Tukey's multiple comparisons test) for the differences between Senexin B-treated and untreated conditions.

The effects of Senexin B on DEGs affected by the first three signals are shown in Figure 5, including serum (added for 30 min following 48 hrs serum starvation) (Figure 5A), NFκB inducer TNF (10 ng/ml for 2 h) (Figure 5B) and PKC agonist, phorbol ester PMA added for 2 h (Figure 5C) or 24 h (Figure 5D). Senexin B-affected DEGs (marked with red dots in Figure 5A–D) comprised 77% of all the genes that were affected by serum, 21% of TNF-regulated genes, 6% of genes affected by 2-h PMA treatment and 3% of genes affected by 24-h PMA treatment. All or almost all of the genes affected both by the signals and by Senexin B were induced by the signals, whereas Senexin B decreased their induction, indicating that Mediator kinase acts primarily as a positive regulator of gene expression induced by these signals. The percentage of Senexin B-regulated DEGs gradually increases if only the top 50%, 20%, 10% or 5% most-strongly signal-induced genes are considered (Figure 5A–D), reaching 100%, 100%, 38% and 36% among the top genes induced by serum, TNF, 2-h PMA and 24-h PMA, respectively. Hence, the genes that are most strongly induced by different signals are also most likely to be affected by Mediator kinase inhibition, indicating that their induction is augmented by CDK8/19.

Figure 5E–H compares the effects of Senexin B on the expression of all the genes that were affected by the CDK8/19 inhibitor either under basal conditions or in the presence of the signals. Red circles mark signal-regulated genes and blue circles mark genes that are not regulated by the signals (many signal-inducible genes were silent in the absence of signals (CPM < 1); such genes are plotted as unaffected by Senexin B under basal conditions). All or almost all the genes affected by short-term Senexin B and signal exposures (Figure 5E–G) were downregulated but most of the genes affected by 24-h treatment were upregulated (Figure 5H). Most of the Senexin B-downregulated genes were affected to a greater degree after signal addition than under the basal conditions, as indicated by the majority of symbols falling below the diagonal in the lower left quadrants (Figure 5E–G), indicating that the positive regulation of gene expression by CDK8/19 is more prominent under the conditions of signal stimulation. Strikingly, the majority of 46 genes that were inhibited by 3-h treatment with Senexin B under basal conditions were induced by serum stimulation or by 2-h PKC induction with PMA (Figure 5I), which affect signals present under basal cell culture conditions (see Discussion), indicating that the early response to CDK8/19 inhibition may primarily reflect the effect on transcription induced by signals in cell culture media. In contrast, many of the late-response genes that were induced by Senexin B after 24 h were no longer induced in the presence of PMA (Figure 5H), indicating that long-term treatment with the PKC agonist broadly altered the negative regulation of gene expression by CDK8/19. As discussed below, this effect may reflect a chromatin rearrangement affecting the distribution of Mediator.

To determine the relative contributions of CDK8 and CDK19 kinase activities to signal-induced gene expression, we have used qPCR to measure the effects of CDK8 and CDK19 reconstitution on the basal and signal-induced expression of selected Senexin B-affected genes that are stimulated by serum (Figure 5J), TNF (Figure 5K) and PMA (Figure 5L). In the absence of active Mediator kinases, almost all the tested genes were still inducible by the corresponding signals but Senexin B had no effect on their induction. Reconstitution of the wild-type (but not mutant) CDK8 or CDK19 in dKO derivatives increased the induction of these genes by PMA and TNF but not by serum, whereas the induction by all the signals, including serum, became susceptible to inhibition by Senexin B. This result indicates that Mediator kinase expression exerts a partial switch from Mediator-kinase independent to Mediator kinase-dependent transcriptional activation mechanisms.

### Effects of CDK8 and CDK19 on IFNγ-regulated transcription and STAT1 S727 phosphorylation

**Figure 6.**
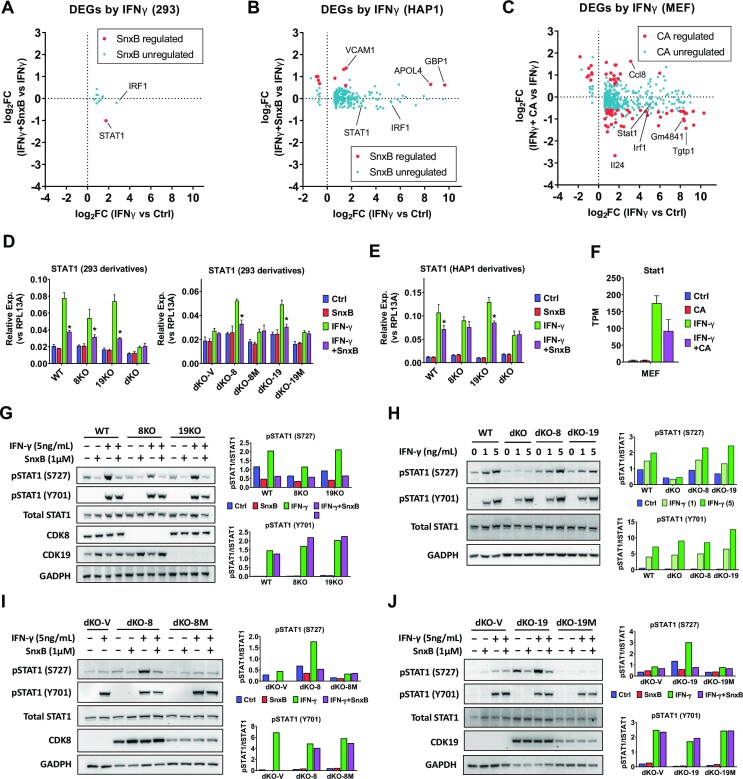
Effects of Mediator kinases on IFNγ-regulated gene expression and STAT1 S727 phosphorylation. (A, B) RNA-Seq analysis of 293 (**A**) and HAP1 cells (**B**) treated with 1 μM Senexin B (SnxB), 10 ng/ml IFNγ (4 h) or SnxB + IFNγ combination (in biological triplicates). The dot plots show the effects of Mediator kinase inhibition on the IFNγ-regulated DEGs (FC > 1.5; FDR < 0.05). Red dots: Senexin B-affected DEGs. Blue dots: Senexin B-unaffected DEGs. (**C**) Analysis of RNA-Seq data from (12) for the effects of cortistatin A (CA) on IFNγ-regulated genes in MEF cells, treated with 100 nM CA, 10 ng/ml IFNγ or CA/ IFNγ combination for 6 h (in biological replicates, *n* ≥ 2). The dot plots show the effects of CA on the IFNγ-regulated DEGs (FC > 1.5; FDR < 0.05). Red dots: CA-affected DEGs. Blue dots: CA-unaffected DEGs. (D, E) qPCR analysis of STAT1 RNA in 293 (**D**) or HAP1 (**E**) derivatives treated with or without 10 ng/ml IFNγ (4 h) or 1 μM Senexin B. Data are presented as mean ± SEM (*n* = 3). Asterisks: *P* < 0.01 (two-way ANOVA, Tukey's multiple comparisons test) for the differences between Senexin B-treated and untreated conditions. (**F**) Expression of STAT1 RNA in MEF cells treated with or without IFNγ (6 h) or CA, presented as mean ± SEM (*n* ≥ 2) based on TPM values of RNA-Seq data from (12). (**G**) Parental (WT) 293 and their 8KO and 19KO derivatives were treated with 1 μM Senexin B, 5 ng/ml IFNγ or Senexin B/IFNγ combination for 5 h and analyzed by immunoblotting for phosphorylated STAT1 (S727 or Y701), STAT1, CDK8, CDK19 and GADPH. (**H**) Parental (WT) 293, and their dKO, dKO-8 and dKO-19 derivatives were treated with 0, 1 and 5 ng/ml recombinant IFNγ for 5 h and analyzed as in (G). (**I**) dKO derivatives dKO-V, dKO-8 and dKO-8M were treated and analyzed as in (G). (**J**) dKO derivatives dKO-V, dKO-19 and dKO-19M were treated and analyzed as in (G). Bar diagrams on the right represent mean densitometry signals from duplicate experiments.

While Mediator kinases are not known to have a direct effect on transcription factors that regulate the induction of gene expression by serum, PMA or TNF, the effect of CDK8/19 on IFNγ-induced transcription has been linked to a direct effect on STAT1, a transcription factor involved in IFNγ response. STAT1 is directly phosphorylated by CDK8 at S727 (8), and this phosphorylation is inducible by IFNγ. STAT1 S727 phosphorylation has become a widely used biomarker of Mediator kinase activity, although it also occurs in the absence of CDK8/19, indicating that this phosphorylation is also induced by other kinases (34). Interestingly, the effects of Senexin B on the transcriptomic effects of IFNγ showed a more complicated pattern than with the other signals. Only 12 genes were induced by 4-h treatment with IFNγ (10 ng/ml) in 293 cells and the induction of only one of them (STAT1) was significantly suppressed by Senexin B (Figure 6A). We therefore analyzed the effects of IFNγ and Senexin B in a known IFNγ-responsive cell line, HAP1 leukemia (39). Many more genes (239) were affected (mostly induced) by 4-hr treatment with IFNγ in HAP1 cells but only 6 IFNγ-induced and 4 IFNγ-inhibited genes were significantly affected by Senexin B, and all such genes were upregulated (Figure 6B). We also analyzed RNA-Seq data of Steinparzer *et al.* (12) on the effects of Mediator kinase inhibitor Cortistatin A (CA) on IFNγ regulated gene expression in MEF (Figure 6C), using the same DEG selection criteria as in our studies (FC > 1.5, FDR < 0.05). In this case, 15% (67 of 440) of IFNγ regulated genes were affected by CA and most of such genes were downregulated by CA and induced by IFNγ. 52% (11 of 21) of the top 5% IFNγ-induced genes were downregulated by CDK8/19 inhibition, resembling the pattern observed in 293 cells with the other signals (Figure 5). Interestingly, STAT1 induction was affected at least to some degree by Mediator kinase inhibition in all three assayed cell lines (Figure 6D–F). It is conceivable that the diverse effects of Mediator kinase inhibition on IFNγ response may reflect the complicated transcriptomic effects of STAT1 and of its phosphorylation at S727 (see Discussion).

Since IFNγ-induced STAT1 S727 phosphorylation was suggested to be mediated by CDK8 but not by CDK19 (12), we have investigated the effects of CDK8 and CDK19 expression on basal and IFNγ-induced STAT1 phosphorylation in 293 cell derivatives. Upon the addition of IFNγ, STAT1 tyrosine (Y701) phosphorylation was induced (from undetectable levels), and serine (S727) phosphorylation was increased relative to the basal level (Figure 6G). S727 phosphorylation (both basal and IFNγ-induced) but not Y701 phosphorylation was inhibited by Senexin B treatment in WT, 8KO and 19KO cells but not in dKO (Figure 6G). Basal and IFNγ-induced S727 phosphorylation was partially decreased by the knockout of CDK8 alone and further decreased in dKO cells but not by the knockout of CDK19 alone (Figure 6G, H). Nevertheless, the reconstitution of either CDK8 or CDK19 (but not their kinase-inactive versions) in dKO cells increased both basal and IFNγ-induced S727 phosphorylation and restored the sensitivity of this phosphorylation to Senexin B (Figure 6H–J), indicating that both CDK8 and CDK19 can phosphorylate STAT1 at S727.

### CDK8/CDK19 ratios account for different effects of CDK8 and CDK19 knockouts on transcription and STAT1 S727 phosphorylation

While our results demonstrate that CDK8 and CDK19 expression have very similar qualitative effects on gene expression and STAT1 S727 phosphorylation, both of these readouts were affected by single knockout of CDK8 whereas CDK19 knockout had only a weak effect. We have asked if this could be due to a mechanistic difference between the functions of CDK8 and CDK19, as previously suggested (12), or to a lower expression of CDK19 relative to CDK8. We have therefore measured the relative CDK8 and CDK19 protein levels in 293 cells. This analysis was carried out by comparing immunoblotting signal intensity of CDK8- and CDK19-specific bands between serial dilutions of 293 whole cell extract and recombinant human CDK8 and CDK19 proteins tagged with GST at their N-termini; immunoblotting for GST was used to normalize the properly sized signals of the recombinant CDK8 and CDK19 proteins (which differ primarily at their C-termini). The results of replicate experiments are shown in Supplementary Figure S6A; the ratio of CDK8 to CDK19 proteins in 293 cell extract was calculated to be 3.0 ± 0.3. The excess of CDK8 over CDK19 can explain why CDK19 knockout has only a minor phenotypic effect in 293 cells.

**Figure 7.**
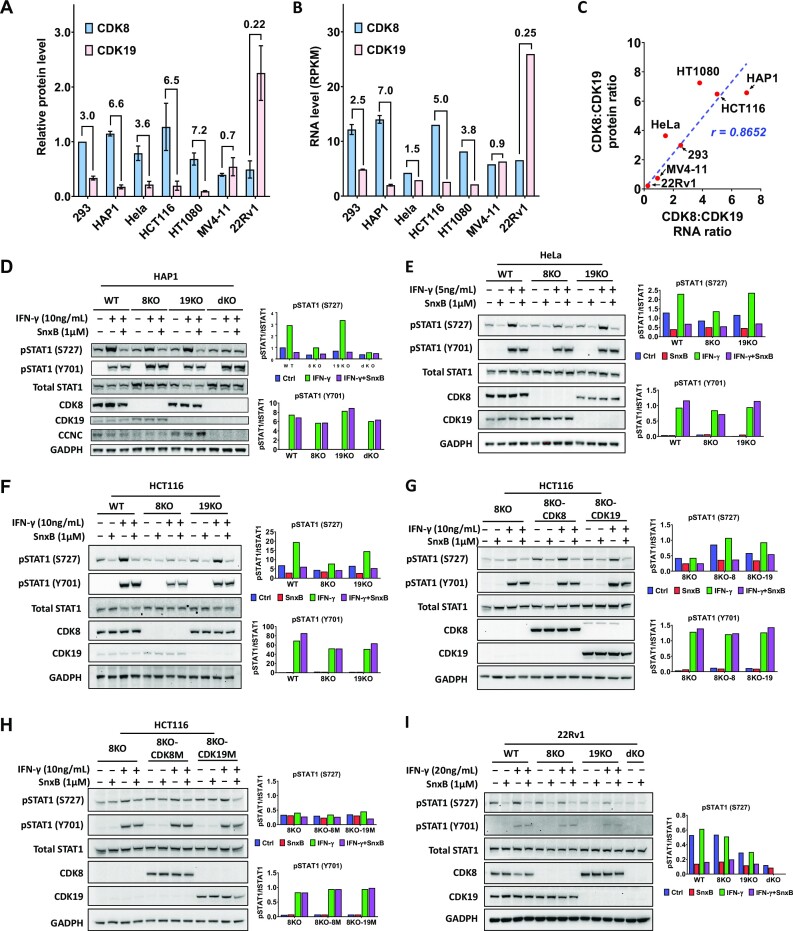
Expression of CDK8 and CDK19 proteins and effects of CDK8 and CDK19 knockout and re-expression on STAT1 S727 phosphorylation in different cell lines. (**A**) Relative protein levels of CDK8 and CDK19, normalized to CDK8 protein level in 293 cells, in different cell lines, determined as shown in Supplementary Figure S6. Data are presented as mean ± SEM of biological triplicates. Ratios of CDK8 to CDK19 for each cell line are shown on top of the bars. (**B**) RNA levels of CDK8 and CDK19 in different cell lines (RPKM, Reads Per Kilobase, per Million mapped reads) from RNA-Seq in this study (293 cells) and from CCLE database (all the other cell lines). (**C**) Correlation of CDK8:CDK19 ratios between RNA and protein levels among different cell lines. (**D**) HAP1 (WT) and their CDK8 or CDK19 single- or double-knockout derivatives (8KO, 19KO, dKO) were treated with 1 μM Senexin B, 10 ng/ml recombinant IFNγ, or Senexin B/IFNγ combination for 5 hrs before immunoblotting analysis for phosphorylated STAT1 (S727 or Y701), STAT1, CDK8, CDK19, CCNC and GADPH. (**E**) HeLa (WT) and their CDK8 or CDK19 single-knockout derivatives (8KO or 19KO) were treated with 1 μM Senexin B, 5 ng/ml recombinant IFNγ, or Senexin B + IFNγ combination for 5 hrs and analyzed as in (D). (**F**) HCT116 (WT) and their CDK8 or CDK19 single-knockout derivative (8KO or 19KO) were treated with 1 μM Senexin B, 10 ng/ml recombinant IFNγ, or Senexin B + IFNγ combination for 1 hr before Immunoblotting analysis. (**G**) HCT116-8KO and their reconstitution derivatives (8KO-8, 8KO-K19) were treated and analyzed as in (D). (**H**) HCT116-8KO and their reconstitution derivatives (8KO-8M, 8KO-19M) were treated and analyzed as in (D). (**I**) 22Rv1 (WT) and their CDK8 and CDK19 single- or double-knockout derivatives (8KO, 19KO, dKO) were treated with 1 μM Senexin B, 20 ng/ml recombinant IFNγ, or Senexin B/IFNγ combination for 5 h and analyzed as in (D). Bar diagrams on the right in (D–I) represent mean densitometry signals from duplicate experiments.

We then compared the relative levels of CDK8 and CDK19 between 293 and several other human cell lines (HeLa cervical carcinoma, HCT116 colon carcinoma, HT1080 fibrosarcoma, MV4-11 acute myeloid leukemia, HAP1 chronic myeloid leukemia), as well as 22Rv1 prostate cancer cell line, which represents the only type of cancer where CDK19 is known to be systematically upregulated (40–43). Using serial dilutions of cell extracts (Supplementary Figure S6B), we found that the ratio of CDK8 to CDK19 was even higher in most of the cell lines than in 293 (7.2 in HT1080, 6.6 in HAP1, 6.5 in HCT116 and 3.6 in HeLa), whereas MV4-11 expressed similar levels of CDK8 and CDK19 (0.9) and 22Rv1 cells expressed 4.5 times more CDK19 than CDK8 (Figure 7A). We have also determined CDK8/CDK19 RNA ratios in the same cell lines using our RNA-Seq data for 293 cells and RNA-Seq data of Cancer Cell Line Encyclopedia (CCLE) for all the other cell lines (Figure 7B). The RNA and protein ratios for CDK8 and CDK19 showed an excellent correlation (*r* = 0.8652) among different cell lines (Figure 7C).

To evaluate the phenotypic effects of CDK8 and CDK19 in other cell lines, we have generated HAP1 leukemia derivatives with the knockout of CDK8, CDK19 or both CDK8 and CDK19 (dKO). In agreement with the predominance of CDK8 in HAP1 cells, only CDK8 but not CDK19 knockout reduced basal and IFNγ-induced STAT1 S727 phosphorylation in HAP1 (Figure 6D). However, the knockout of CDK8 alone did not reduce STAT1 S727 phosphorylation to the level of dKO and did not completely abolish the inhibitory effect of Senexin B. As in the case of 293 cells, CCNC levels of HAP1 were reduced by CDK8 knockout and further decreased by dKO (Figure 7D). Similarly, only CDK8 knockout significantly reduced basal and IFNγ-induced STAT1 S727 phosphorylation in HeLa (Figure 7E) and HCT116 (Figure 7F) cells but the knockout could not fully abolish the inhibitory effect of Senexin B. To confirm that both CDK8 and CDK19 can induce STAT1 S727 phosphorylation in HCT116, we expressed WT or kinase-inactive CDK8 or CDK19 in HCT116 cells with CDK8 knockout. Both WT CDK8 and CDK19 enhanced basal and IFNγ-induced STAT1 S727 phosphorylation (Figure 7G, H). As expected, kinase-inactive CDK8 or CDK19 showed no effect.

We also generated derivatives of 22Rv1 prostate cancer cells (which overexpress CDK19 relative to CDK8) with the knockout of CDK8 and CDK19, individually and in combination (dKO), and analyzed STAT1 S727 phosphorylation with and without IFNγ treatment (Figure 7I). Interestingly, this cell line showed only very weak induction of STAT1 Y701 phosphorylation even by a high dose of IFNγ (20 ng/ml), with no significant increase in STAT1 S727 phosphorylation. Basal STAT1 S727 phosphorylation in 22Rv1 cells, however, was quite prominent and it was strongly inhibited by Senexin B or by dKO. In contrast to the other tested cell lines, the knockout of CDK19 alone in 22Rv1 cells had a stronger effect on STAT1 S727 phosphorylation than CDK8 knockout, in agreement with the high ratio of CDK19 to CDK8. These results demonstrate that differential effects of CDK8 and CDK19 depletion on basal and signal-induced STAT1 S727 phosphorylation are determined by differences in relative protein expression rather than qualitative differences between the functions of these two paralogs.

**Figure 8.**
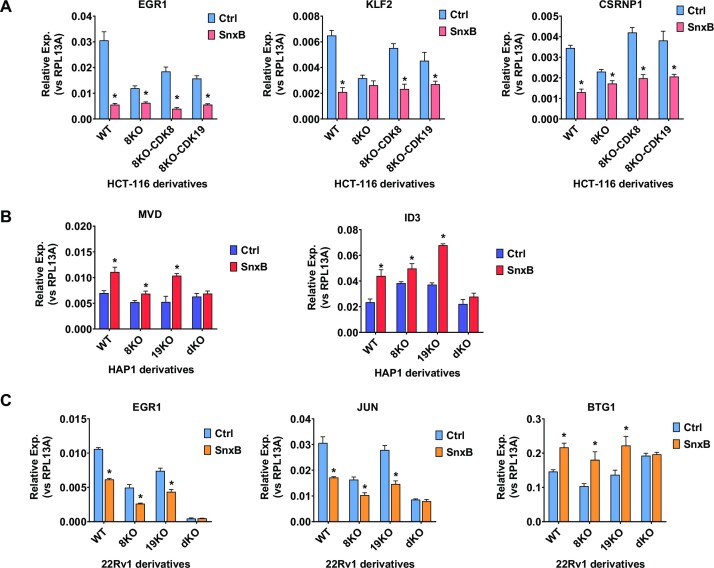
Effects of CDK8/19 inhibitor treatment and CDK8/19 knockout or expression on gene expression in different cell lines. (**A**) HCT116 (WT) cells and their 8KO, 8KO-8 and 8KO-19 derivatives were treated with or without 1 μM Senexin B for 5 h before RNA extraction and qPCR analysis of EGR1, KLF2 and CSRNP1 mRNA. (**B**) HAP1 (WT) cells and their 8KO, 19KO and dKO derivatives were treated with or without 1 μM Senexin B for 5 h before RNA extraction and qPCR analysis of MVD and ID3 mRNA. (**C**) 22Rv1 (WT) cells and their CDK8 8KO, 19KO and dKO derivatives were treated with or without 1 μM Senexin B for 24 hrs before RNA extraction and qPCR analysis of EGR1, JUN and BTG1 mRNA. Data are presented as mean ± SEM (*n* = 3). Asterisks: *P* < 0.01 (two-way ANOVA, Tukey's multiple comparisons test) for the differences between Senexin B-treated and untreated conditions.

We also extended our analysis of the effects of CDK8 and CDK19 on gene expression beyond 293 cells, using qPCR to analyze the effects of CDK8 and CDK19 modifications on the expression of Senexin B-regulated genes in different cell lines. Figure 8A shows that the knockout of CDK8 in HCT116 cells (CDK8/CDK19 ratio 6.5) largely decreases both the expression and the effect of Senexin B on EGR1, KLF2 and CSRNP1. However, the expression of either CDK8 or CDK19 in the CDK8 knockout HCT116 cells restores the expression and Senexin B regulation of these genes. Figure 8B shows that the induction of MVD and ID3 by Senexin B in HAP1 cells (CDK8/CDK19 ratio 6.6) is greatly (but not completely) diminished by the knockout of CDK8 but not CDK19, whereas the knockout of both CDK8 and CDK19 abolishes the induction. Figure 8C shows that the knockout of either CDK8 or CDK19 alone in 22Rv1 cells (CDK8/CDK19 ratio 0.22) does not prevent the inhibition of EGR1 or JUN or the induction of BTG1 by Senexin B, but this response is fully abolished in dKO derivatives. Hence, both CDK8 and CDK19 regulate gene expression in different cell types.

### Proteomic analysis reveals negative post-transcriptional regulation of mediator complex components by CDK8 and CDK19 kinases

**Figure 9.**
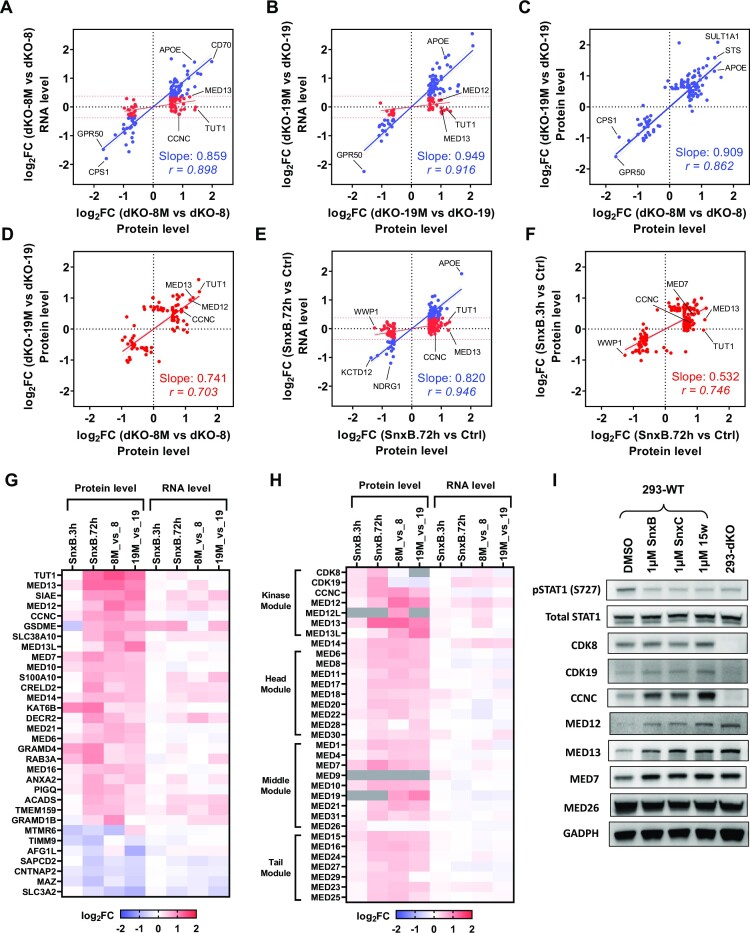
Proteomic analysis of the effects of CDK8 and CDK19 kinase inhibition. Tandem Mass Tag (TMT) based proteomic analysis was carried out for the effects of CDK8 and CDK19 kinase inhibition across three TMT-11plex batches: dKO-8 vs dKO-8M, dKO-19 versus dKO-19M and parental 293 cells treated with DMSO (Ctrl), Senexin B (3 h) or Senexin B (72 h). (**A**) Comparison of the effects of CDK8 kinase domain mutation (dKO-8M versus dKO-8) on the RNA and protein levels for the genes differentially expressed at the protein level. Red dots: genes whose RNA expression levels differ <1.3-fold. Blue dots: genes whose RNA expression levels differ ≥1.3-fold. (**B**) Comparison of the effects of CDK19 kinase domain mutation (dKO-19M versus dKO-19) on the RNA and protein levels as in (A). (**C**) Comparison of the effects of CDK8 and CDK19 kinase domain mutations on the expression of proteins affected ≥1.3-fold at the RNA level. (**D**) Comparison of the effects of CDK8 and CDK19 kinase domain mutations on the expression of proteins affected <1.3-fold at the RNA level. (**E**) Comparison of the effects of 72-h treatment with Senexin B on the RNA and protein levels as in (A). (**F**) Comparison of the effects of 3- and 72-h Senexin B treatment for the proteins affected <1.3-fold at the RNA level. Slope and Pearson correlation coefficients (*r*) in were calculated by linear regression and correlation analysis for (A–F). (**G**) Heatmap of the effects of Senexin B treatment (3 or 72 h) and CDK8 or CDK19 kinase domain mutations on the protein and RNA levels for the genes regulated by CDK8/19 kinase activity at the post-transcriptional level and detected in all the protein batch comparisons. (**H**) The same heatmap for all the components of the kinase module and the core Mediator complex (grey: protein not detected). (**I**) Expression of the indicated proteins in parental 293 cells, untreated or treated with 1 μM Senexin B, Senexin C or 15w for 24 h and in untreated 293-dKO cells.

To determine how the transcriptomic effects of CDK8 and CDK19 correlate with their proteomic effects, we have carried out Tandem Mass Tag (TMT) based proteomic and phosphoproteomic analysis of the effects of CDK8 and CDK19 in 293 cells (without signal stimulation). A total of 30 samples were multiplexed across three TMT-11plex batches. The first batch included dKO-8 and dKO-8M (five biological replicates of each), the second included dKO-19 vs dKO-19M (5 + 5 replicates), and the third included parental 293 cells treated with DMSO (Ctrl), Senexin B (3 h) or Senexin B (72 hrs) (4 + 3 + 4 replicates, correspondingly). Data dependent acquisition was used to quantitate peptides from three separate batches of TMT multiplexed samples, which introduces a TMT batch effect that caused a subset of proteins and phosphoepitopes not to be identified in all batches. Only proteins detected in all the samples compared were used for the analysis shown in Figure 9. Proteins affected by Senexin B treatment or Mediator kinase mutations are listed in Supplementary Table S6.

A total of 226 proteins, selected by the criteria FC > 1.5, FDR < 0.05, were differentially expressed in the presence of kinase-active or inactive forms of CDK8 (dKO-8 versus dKO-8M, 151 out of 7460 detected proteins) or CDK19 (dKO-19 versus dKO-19M, 125 out of 7447 proteins). The effects of the kinase domain mutations on the expression of the corresponding genes at the RNA and protein levels are compared in Figure 9A (for CDK8) and Figure 9B (for CDK19). This analysis distinguished between two sets of proteins that either were or were not regulated at the RNA level (based on RNA-Seq analysis, FC < 1.3 was chosen as the cutoff for lack of RNA regulation). For the genes that are upregulated or downregulated at the RNA level (blue dots in Figure 9A, B), the effects of the kinase mutations on the RNA and protein levels show excellent correlations for both CDK8 and CDK19. Most of the proteins that were not regulated at the RNA level (red dots in Figure 9A, B) were upregulated by the expression of mutant over WT Mediator kinases, including 55 of 74 CDK8-regulated proteins and 28 of 41 CDK19-regulated proteins. The effects of CDK8 and CDK19 kinase domain mutations on the expression of proteins regulated at both protein and RNA levels are very strongly correlated with each other (Figure 9C). The correlation between the effects of CDK8 and CDK19 is also pronounced but not as strong for proteins that are regulated at the protein but not the RNA level (Figure 9D).

A total of 238 proteins (out of 7125 detected) were regulated by Senexin B at 3 h (59 proteins) or 72 h of treatment (212 proteins). Only 67 of 212 proteins were affected by 72-h Senexin B treatment at the RNA level with FC > 1.3 (blue dots in Figure 9E), and the effects of Senexin B on such proteins were well correlated at the RNA and protein levels. Only 1 of 59 detected proteins impacted by 3-h Senexin B treatment was affected at both RNA and protein levels, and most of the proteins regulated by 72-h Senexin B treatment were not regulated at the RNA level (red dots in Figure 9E). Post-transcriptional effects of Senexin B were much greater at the 72-h than at 3-h point, as indicated both by the 3 times higher number of affected proteins and by the stronger effect of 72-h treatment on proteins affected at both time points (Figure 9F). Figure 9G shows a heat map of the effects of Senexin B treatment (3 or 72 h) and CDK8 or CDK19 kinase domain mutations on the levels of proteins that were detected in both types of analysis and regulated by CDK8/19 kinase activity at post-transcriptional level. 25 of 32 proteins in this group were upregulated both by Senexin B treatment and by kinase domain mutations. Remarkably, 40% of these proteins were components of Mediator kinase module or the core Mediator complex. Figure 9H shows the effects on all the 33 proteins comprising the kinase module or the head, middle and tail modules of the core Mediator complex. Strikingly, Mediator kinase inhibition leads to stabilization of all the Mediator proteins, except for MED26, the Mediator complex subunit that was reported to be excluded from core Mediator when it is bound to Mediator kinase module (44) and therefore does not associate with CDK8/19. None of the Mediator subunits show comparable regulation by CDK8/19 inhibition at the RNA level, although CDK19, MED12 and MED14 RNA were slightly upregulated (Figure 9H).

The results of proteomic analysis agree with immunoblotting results in Figure 1B that showed CCNC, MED12 and MED13 to be upregulated by CDK8 or CDK19 kinase domain mutations. To determine if the increase in protein levels of the Mediator kinase module and core Mediator subunits is a general consequence of CDK8/19 inhibition, we have treated parental 293 cells for 24 hrs with 1 μM concentrations of different CDK8/19 inhibitors including 15w, Senexin C (35) and Senexin B. Figure 9I shows that all three compounds decrease STAT1 S727 phosphorylation while at the same time increasing the protein levels of CCNC, MED12, MED13 and MED7 but not MED26, in agreement with the proteomic data. MED12, MED13 and MED7 are also upregulated in dKO cells, where CCNC is degraded (Figure 9I). Hence, CDK8/19 kinase activity exerts a negative post-transcriptional regulation on the components of both the CDK module and the core Mediator complexes. Upregulation of this transcriptional complex increases over the time of CDK8/19 inhibition (Figure 9H), offering an explanation for the delayed induction of transcription by CDK8/19 inhibitor treatment.

### Phosphoproteomic analysis of the effects of CDK8 and CDK19 kinase inhibition

**Figure 10.**
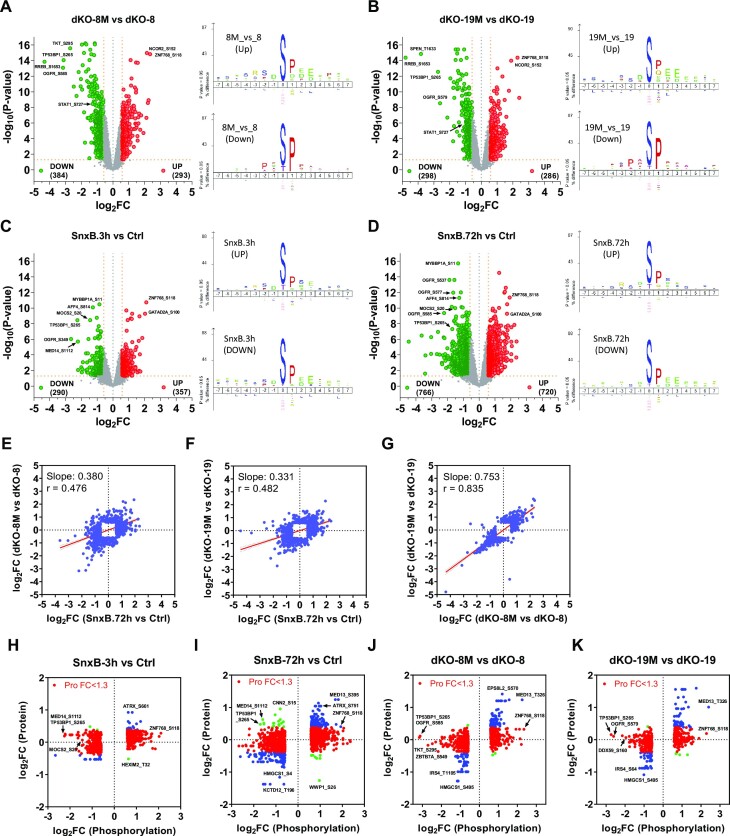
Phosphoproteomic analysis of the effects of CDK8 and CDK19 kinase inhibition. Tandem Mass Tag (TMT) based phosphoproteomic analysis was carried out for the effects of CDK8 and CDK19 kinase inhibition across the same three TMT-11plex batches as in Figure 9. (A–D) Volcano plots of phosphoepitope changes (left) and results of motif enrichment analysis (right) for the comparisons of dKO-8M versus dKO-8 (**A**), dKO-19M versus dKO-19 (**B**), 3-h Senexin B treatment versus control (**C**), and 72-h Senexin B treatment versus control (**D**). (**E**) Comparison of the effects of CDK8 kinase domain mutation and 72-h Senexin B treatment on protein phosphorylation. (**F**) Comparison of the effects of CDK19 kinase domain mutation and 72-h Senexin B treatment on protein phosphorylation. (**G**) Comparison of the effects of CDK8 and CDK19 kinase domain mutations on protein phosphorylation. (H–K) Comparisons of fold changes in the protein levels to changes in phosphorylation for differentially expressed phosphoepitopes after 3-h (**H**) or 72-h Senexin B treatment (**I**), in cells expressing kinase-inactive CDK8 mutant versus WT CDK8 (**J**) or kinase-inactive CDK19 mutant versus WT CDK19 (**K**). Red dots: phosphoproteins affected with FC < 1.3 at the protein level. Blue dots: phosphoproteins affected with FC ≥ 1.3 at the protein level in the same direction as phosphoprotein changes. Green dots: phosphoproteins affected with FC ≥ 1.3 at the protein level in the opposite direction to phosphoprotein changes (H–K).

We have carried out phosphoproteomic analysis of TMT data to compare dKO cells expressing WT or kinase-inactive forms of CDK8 or CDK19. Phosphoepitopes affected by Senexin B or Mediator kinase mutations (FC > 1.5, FDR < 0.05) are listed in Supplementary Table S7. Figure 10A–D shows volcano plots and enriched motif analysis using iceLogo (45) for phosphoepitopes regulated by CDK8 kinase activity (Figure 10A), by CDK19 kinase activity (Figure 10B), 3-hr treatment (Figure 10C), and 72-hr treatment with Senexin B (Figure 10D). An S/T-P enriched motif similar to the one previously associated with Mediator kinase inhibition in HCT116 cells (46) was detected in all four comparisons of phosphoepitopes downregulated by CDK8/19 inhibition. The effects of CDK8 and CDK19 kinase domain mutations correlate with the effects of 72-hr Senexin B treatment of parental cells (Figure 10E, F). The phosphoproteomic effects of CDK8 and CDK19 are very strongly correlated with each other (Figure 10G). Together with the results of STAT1 S727 phosphorylation analysis, the phosphoproteomic data reveal that CDK8 and CDK19 have qualitatively the same effect on protein phosphorylation.

We have asked if phosphoepitope changes could be due to changes in the total protein levels or, conversely, if proteomic changes could reflect changes in protein stability consequential to CDK8/19-mediated phosphorylation. Figure 10H–K compare fold changes in the protein levels to changes in the expression of the most strongly affected phosphoepitopes for the same protein. In these plots, red dots mark phosphoproteins that were affected with FC < 1.3 at the protein level, blue dots mark phosphoproteins affected with FC > 1.3 at the protein level in the same direction as phosphoprotein changes, and green dots mark phosphoproteins affected with FC > 1.3 at the protein level in the opposite direction to phosphoprotein changes. Most of the affected phosphoproteins were not altered >1.3-fold at the protein level (red dots), suggesting that the observed effects on such proteins were at the level of phosphorylation. Among proteins affected at both proteomic and phosphoproteomic levels, the majority showed changes in the same direction by both parameters (blue dots). A few proteins showed opposite directions of proteomic and phosphoproteomic changes, including MED14 (phosphorylated at S1112) and TP53BP1 (phosphorylated at S265), phosphorylation of which was strongly decreased both after 3 or 72 h of Senexin B treatment, while the levels of these proteins became increased only after 72 h treatment. This pattern suggests that such proteins could be destabilized by CDK8/19-mediated phosphorylation.

**Figure 11.**
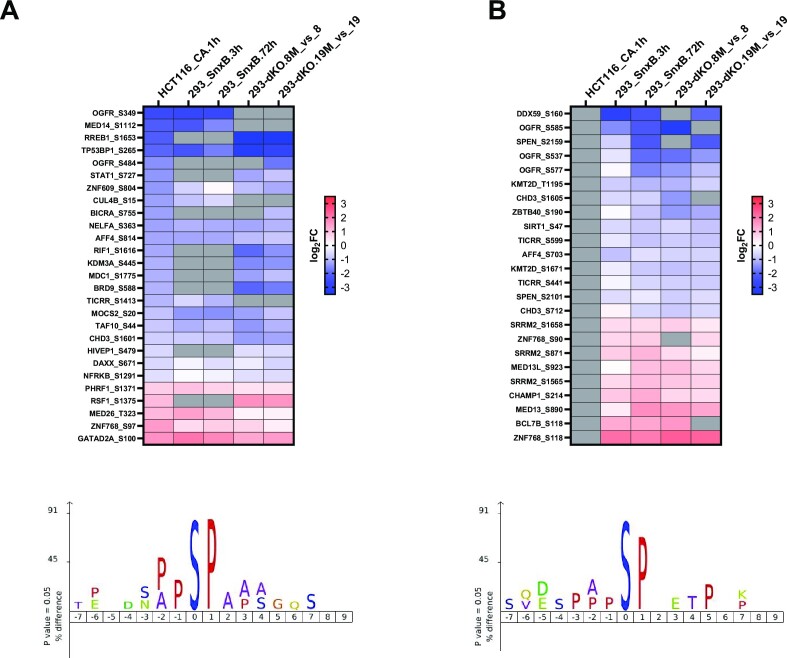
Comparative phosphoproteomics analysis of the effects of Mediator kinase inhibition in 293 and HCT116 cells. (**A**) Heatmap of the 27 phosphoepitopes similarly affected by Mediator kinase inhibition in HCT116 (48) and 293 cells (this study). (**B**) Heatmap of 24 representative phosphoepitopes (discovered in at least three comparisons in 293 cells) that were not identified in HCT116 study (48). Enriched motif analysis of the downregulated phosphoepitopes is presented below the heatmaps.

We have also compared the results of our phosphoproteomic analysis of CDK8/19 kinase domain mutations or Senexin B treatment of 293 cells with the data of Poss *et al.* (46) based on 1-h CA treatment of HCT116 cells (Supplementary Table S8). Among 75 proteins whose phosphorylation was affected by CA treatment in HCT116 cells, 59 proteins were detected in our study and 49 of them (83%) were also affected by CDK8/19 kinase activity (observed in at least one of the comparisons: dKO-8M vs dKO-8, dKO-19M vs dKO-19, WT-Senexin B_3h and WT-Senexin B_72h). Among the 64 phosphoepitopes affected in 49 proteins in Poss *et al.* (46), 37 phosphoepitopes (33 proteins) were detected in our study. 27 phosphoepitopes (26 proteins) were similarly affected in both studies (Figure 11A), suggesting that such phosphoepitopes could potentially provide biomarkers of Mediator kinase activity in different cell types. The commonly affected phosphoepitopes are found in nuclear phosphoproteins OGFR-S349, MED14-S1112, RREB1-S1653, TP53BP1-S265, STAT1-S727, NELFA-S363, AFF4-S814, BRD9-S588, TAF10-S44 and CHD3-S1601, phosphorylation of which is reduced by Mediator kinase inhibition, as well as MED26-T323, ZNF768-S97 and GATAD2A-S100, phosphorylation of which is increased by Mediator kinase inhibition. Figure 11B shows a heat map of 24 representative phosphoepitopes (discovered in at least three comparisons) that were not identified in the study on HCT116. Enriched motif analysis of the downregulated phosphoepitopes in Figure 10A and B showed very similar (P/A)PSP motives, suggesting that the newly identified downregulated phosphoepitopes are likely to be Mediator kinase phosphorylation substrates.

## DISCUSSION

### Functional similarity of CDK8 and CDK19 and limitations of mediator kinase knockout models

We have carried out a detailed transcriptomic, proteomic and phosphoproteomic analysis to elucidate the functions of CDK8 and CDK19 Mediator kinases. In this analysis, we did not base our conclusions on comparisons between parental cells and CDK8/19 knockout clones, which are affected by clonal variability and by target-independent effects of sgRNA/CRISPR. Instead, our conclusions relied initially on comparisons between mass populations of cells with the knockout of both CDK8 and CDK19 followed by reconstitution with either WT or kinase-inactive CDK8 or CDK19 proteins. CDK8 or CDK19 were overexpressed in such derivatives relative to parental cells, which could have affected the phenotypic outcomes. However, we found that overexpression of WT Mediator kinases in parental cells had no effects on gene expression in 293 cells, our primary model, indicating that the endogenous Mediator kinase levels were sufficient for the maximal transcriptomic effect and were not further affected by an increase in CDK8 or CDK19. Much of our analysis also relied on a CDK8/19-specific kinase inhibitor (Senexin B), the effects of which were confirmed by the concordance with several other selective Mediator kinase inhibitors and by the lack of an effect on cells with the knockout of both CDK8 and CDK19, as well as a novel CDK8/19-degrading PROTAC.

The effects of CDK8 and CDK19 on basal gene expression at the RNA and protein levels and on the phosphorylation of STAT1 S727 and other protein substrates were found to be very similar, although the effects of CDK8 were quantitatively stronger than those of CDK19. The complementary roles of CDK8 and CDK19 were also indicated by the findings reported here and in the literature (2,10,47) that the knockout of both CDK8 and CDK19 (or their common binding partner CCNC) was required to fully mimic the effects of CDK8/19 kinase inhibitors, whereas the knockout of CDK8 or CDK19 alone had at most a partial effect. These results indicate that pharmacological Mediator kinase inhibitors should generally inhibit both CDK8 and CDK19 to achieve therapeutically relevant effects.

While the knockout of both CDK8 and CDK19 (dKO) reproduced most of the effects of the Mediator kinase inhibitors, dKO cells also displayed specific changes that limit the utility of such cells for modelling the effects of the inhibitors. In addition to transcriptomic changes reflecting clonal variability due to the clonal nature of dKO, a subset of genes affected by CDK8/19 inhibitors or the CDK8/19-degrading PROTAC were unaffected by dKO, likely reflecting compensatory changes that arose during the generation of dKO cells. dKO cells also showed strong induction of serum- and NFκB-responsive genes, induction of which was suppressed by CDK8/19 inhibitors in parental cells, indicating a switch from Mediator kinase-dependent to other mechanisms of regulation of such genes. Interestingly, CDK8/19 dependence of signal-responsive gene expression was restored by the expression of CDK8 or CDK19 in dKO cells.

A prominent effect of dKO (which is also reproduced by the PROTAC) is the degradation of CCNC, the binding partner of CDK8 and CDK19. CCNC degradation was proteasome-dependent and prevented by the expression of wild-type or kinase-inactive CDK8 or CDK19, in agreement with an earlier report that CDK8 can protect CCNC from proteolysis (33). CCNC degradation in dKO cells is likely to have physiological consequences, since CCNC has activities unrelated to the Mediator kinase. In particular, CCNC was reported to bind CDK3 regulating G0 to G1 transition (48). CCNC also plays a Mediator-independent role in mitochondria, where it binds to Drp1 GTPase in the outer membrane, promoting mitochondrial fission and stimulating oxidative stress-induced apoptosis (49). The consequences of CCNC degradation should be taken into account when interpreting the phenotypic effects of CDK8 and CDK19 depletion in cells and organisms.

Due to the complementary functions of CDK8 and CDK19, special care should be taken when interpreting the results of the knockout of individual Mediator kinases. We have observed that the knockout of CDK8 alone diminished (but did not abolish) the effects of the CDK8/19 inhibitor on gene expression and STAT1 S727 phosphorylation, whereas the knockout of CDK19 alone had little effect on these phenotypic readouts. The latter finding resembles the results of Steinparzer *et al.* (12) with siRNA knockdown of CDK19 in MEF. Although the effects of CDK8 on gene expression (Figures 2F, G and 3D, E) and protein phosphorylation (Figure 10G) were quantitatively stronger than those of CDK19, this difference seemed insufficient to account for the very different effects of CDK8 and CDK19 knockouts. Having determined the relative abundance of CDK8 and CDK19 proteins in multiple cell lines, we concluded that all the cell lines (293, HCT116, HeLa, HAP1) where the knockout of CDK8 but not CDK19 alone had a strong effect on transcription and STAT1 S727 phosphorylation expressed greater levels of CDK8 than CDK19. In contrast, CDK19 knockout had a stronger effect than CDK8 knockout on STAT1 S727 phosphorylation and a similar effect on gene expression in 22Rv1 prostate carcinoma cells that overexpress CDK19 relative to CDK8. In addition, STAT1 S727 phosphorylation and CDK8/19-regulated gene expression in HCT116 cells with CDK8 knockout were restored by the expression of either CDK8 or CDK19. Based on these results, along with proteomic and phosphoproteomic studies, we conclude that the differences in the effects of CDK8 and CDK19 on basal gene expression and protein phosphorylation are dictated primarily by differences in their expression and secondarily by quantitative differences in their activity, but not by a qualitative difference in their functions, at least in the cellular systems that we have analyzed.

### Transcriptional and post-transcriptional regulation by CDK8/19 is kinase-dependent

Several studies concluded the existence of kinase-independent phenotypic activities for both CDK8 (14,15) and CDK19 (12,16). As discussed above, CDK8/19 knockout or degradation lead to the degradation of CCNC, which is likely to have phenotypic consequences distinct from those of CDK8/19 kinase inhibition. We have searched more broadly for kinase-independent functions of CDK8 and CDK19, by comparing the effects of their WT and kinase-inactive versions and the effects of a CDK8/19-degrading PROTAC and its cognate CDK8/19 kinase inhibitor. Detailed transcriptomic analysis revealed that kinase-inactive CDK8 and especially CDK19 mutants, when expressed in the parental 293 cells, had a substantial dominant negative effect on gene expression mediated by the WT proteins, with CDK19 mutant producing a stronger effect. However, kinase-inactive CDK8 or CDK19 mutants expressed in dKO cells showed only weak effects on the expression of just a few genes. Neither these nor any other genes were differentially affected by the PROTAC and kinase inhibitor in a CDK8/19-dependent manner, indicating the lack of kinase-independent effects of CDK8/19 on gene expression in the studied cellular models. In addition, CDK8/19 knockout and mutational or pharmacological inhibition of its kinase activity had a similar post-transcriptional effect on the Mediator complex components and other post-transcriptionally regulated proteins, indicating that the post-transcriptional effects were also kinase-dependent.

### Mediator kinases potentiate transcription induced by most signals

The primary effect of CDK8/19 inhibition on gene expression in cells exposed to serum, NFκB inducer TNF, and PKC agonist PMA was the reduction of signal-induced gene expression. CDK8/19 inhibition had the greatest effect on the most strongly signal-induced genes, indicating that Mediator kinases act as positive co-factors amplifying the effects of signal-activated transcription factors. Among the genes that were downregulated by CDK8/19 inhibitor under basal or signal-stimulated conditions, the majority were inhibited to a greater degree in the presence than in the absence of the signal, including many signal-inducible genes that were ‘silent’ (expressed at very low levels) in unstimulated cells. This analysis confirms and extends our previous conclusion, based on the analysis of a small number of signal-stimulated genes (10), that Mediator kinase is a pleiotropic regulator of signal-stimulated transcriptional reprogramming. This function of Mediator kinase is not limited to multicellular organisms, as a recent study in yeast has also concluded that CDK8 kinase activity is required for gene activation under stress but not under steady-state growth conditions (50). Similar positive co-regulation of three different signals by CDK8/19 is likely consequential to the regulation of Pol II CTD phosphorylation in the selective context of signal-activated genes, as previously demonstrated for the serum response network (17), NFκB (10), HIF1α (6) and ER (9).

The effects of CDK8/19 on IFNγ signaling were more complicated. Mediator kinase inhibition did not show preferential suppression of IFNγ induced gene expression in human 293 or HAP1 cells. However, analysis of the RNA-Seq data of Steinparzer *et al.* (12) showed that the CDK8/19 inhibitor preferentially downregulated IFNγ-inducible genes in MEF cells, resembling our results with the other signals in 293 cells. IFNγ signaling is regulated to a large extent by STAT1, which is directly (but not exclusively (34)) phosphorylated by Mediator kinase at S727. STAT1 S727 phosphorylation modulates rather than merely activates STAT1 activity (12,51), which may explain the complicated effects of CDK8/19 inhibition on IFNγ-regulated transcription and the differences between its effects in different cell types. Furthermore, we found that IFNγ induced (but not basal) STAT1 RNA expression was reduced to some extent by Mediator kinase inhibition in all three tested cell lines, suggesting that some of the effects of Mediator kinase on IFNγ regulated genes could be due to the reduced expression of this transcription factor.

### Early and late responses to CDK8/19 inhibition: relation to signal stimulation and to post-transcriptional upregulation of the mediator complex

Under basal cell culture conditions, CDK8/19 inhibitor treatment affected only a small number of genes at an early (3 hrs) time point (46 genes by our cutoff criteria), and all these genes were downregulated. Remarkably, most of these early-response genes were induced upon short-term serum stimulation or 2-h treatment with a PKC agonist PMA (Figure 5I). Serum stimulation and PMA addition mimic signals that are present and have fluctuating activity in conventional cell culture media. In particular, PKC signaling (which is activated by PMA) is controlled by fluctuation in diacylglycerol and Ca levels and interactions with proteins that regulate its activity and stability, through elaborate feedback mechanisms (52). This suggests that most if not all the early responses to Mediator kinase inhibition may be mediated by transcription-stimulating signals present in cell culture, which are positively regulated by CDK8/19.

In contrast to the early inhibition of gene expression, the primary effect of prolonged CDK8/19 inhibitor treatment was the upregulation of a larger number of genes (∼400 by our cutoff criteria), and this upregulation was also observed upon long-term genetic inactivation of the Mediator kinase. Surprisingly, we found that prolonged CDK8/19 inhibition or mutagenesis of the kinase domain not only upregulated a set of genes at the RNA level but also induced post-transcriptional upregulation of a group of proteins, most of which directly or indirectly interact with CDK8/19. Interestingly, one of these proteins is TUT1 implicated in nucleolar integrity (53), a process that we found to be regulated by CDK8 via its interaction with p21 (CDKN1A) (54). The largest group of post-transcriptionally upregulated proteins comprises almost all the components of Mediator complex, with a notable exception of MED26, which is displaced from the Mediator by the Mediator kinase module (44). The increased levels of Mediator, a coactivator of transcription (55), can explain why the late response to CDK8/19 inhibition comprises upregulation of a relatively large set of genes. Furthermore, this result can explain why CDK8/19 inhibition in leukemia cells increased the expression of genes associated with super-enhancers (which are characterized by increased Mediator binding), leading to leukemia suppression (13).

Remarkably, long-term (24 h) treatment of cells with a PKC agonist had a drastic effect on late-response genes that were upregulated by CDK8/19 inhibition under basal conditions, as most of such genes were no longer induced by Senexin B in the presence of PMA (Figure 5H). We hypothesize that this drastic change reflects PMA-induced chromatin rearrangement that includes redistribution of Mediator complexes, which regulate the genes that are upregulated by CDK8/19 inhibition. The association of Mediator with the late-response genes remains to be tested in future studies.

### Direct and indirect effects of CDK8/19 on protein phosphorylation and stability

Inhibition of Mediator kinase activity affected hundreds of phosphoepitopes. Remarkably, similar numbers of proteins showed either decreased or increased phosphorylation upon CDK8/19 inhibition, even after the shortest period of inhibitor treatment (3 h), indicating that many and probably most of the phosphoproteomic effects were indirect. Our phosphoproteomic analysis was not aimed at identifying direct Mediator kinase phosphorylation targets, but enriched motif analysis of the phosphoepitopes that were found here to be downregulated by CDK8/19 inhibition matches the previously identified CDK8/19 phosphorylation motifs (46), suggesting that many of these could be direct Mediator kinase substrates. Furthermore, a number of phosphoproteins affected by CDK8/19 inhibition in 293 cells (such as OGFR, MED14, RREB1, TP53BP1, NELFA, AFF4, BRD9, TAF10, CHD3 and STAT1) were previously identified as likely targets of Mediator kinase in HCT116 colon carcinoma (46). These phosphoepitopes could potentially be used as general markers of CDK8/19 activity in different cell types, but unfortunately antibodies specific to Mediator kinase-regulated phosphoepitopes identified here are not currently available.

Post-transcriptional negative regulation of Mediator kinase-interactive proteins by Mediator kinase activity seems unlikely to be exerted through protein synthesis, given the nuclear localization of CDK8/19. Alternatively, this effect could be mediated by the enhancement of protein degradation (which has not been directly tested in this study). We have asked if protein phosphorylation by CDK8/19 could be responsible for changes in the protein levels, via stabilization or destabilization of the phosphorylated proteins. Consistently with this hypothesis, several proteins (such as TP53BP1 and MED14) showed a strong decrease in phosphorylation after 3-hr treatment with a Mediator kinase inhibitor, followed by an increase in protein levels at the 72-hr treatment point, whereas several proteins (including MED13) showed the same direction of changes in their phosphorylation and expression upon CDK8/19 inhibition. On the other hand, most of the proteins that showed post-transcriptional regulation by Mediator kinase were not identified as CDK8/19-affected phosphoproteins, suggesting that their regulation is more likely to be mediated by differential protein-protein interactions of kinase-active versus inactive CDK8/19.

## Supplementary Material

gkad538_Supplemental_Files

## Data Availability

The RNA-Seq data are available in GEO database (accession numbers GSE154357, GSE149432, GSE101629, GSE199331, GSE200523, GSE200732, GSE200735, GSE200747, GSE221160, GSE200753 and GSE231891). The proteomics and phosphoproteomics data are available in MassIVE (accession number MSV000089494). All the source codes (R scripts) and raw data tables (both processed RNASeq and proteomics/phosphoproteomics data) used for data analysis and plotting in this manuscript are included in the supplemental file named ‘Source Codes and RawData Files.zip’.
