# Supplementary material for: CDK8 and CDK19: positive regulators of signal-induced transcription and negative regulators of Mediator complex proteins
Source: Nucleic Acids Res. 2023 Jun 28;51(14):7288–313. doi: 10.1093/nar/gkad538 (PMC10415139; doi:10.1093/nar/gkad538)
Supplement: gkad538_Supplemental_Files [file gkad538_supplemental_files.zip › NAR_Supplemental_Figures_20230601.pdf]

Figure S1

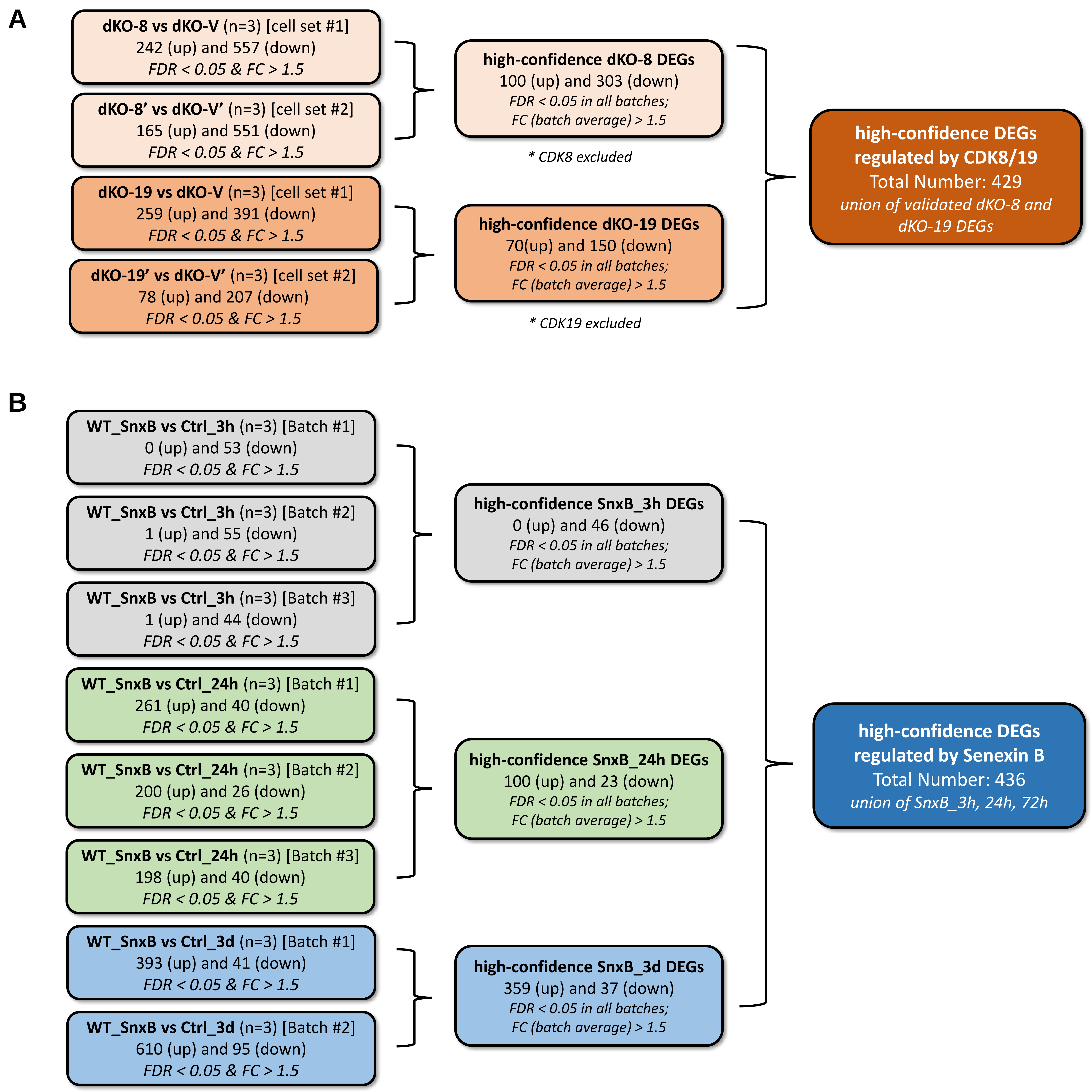
A
dKO-8 vs dKO-V (n=3) [cell set #1]
242 (up) and 557 (down)
FDR < 0.05 & FC > 1.5
dKO-8' vs dKO-V' (n=3) [cell set #2]
165 (up) and 551 (down)
FDR < 0.05 & FC > 1.5
dKO-19 vs dKO-V (n=3) [cell set #1]
259 (up) and 391 (down)
FDR < 0.05 & FC > 1.5
dKO-19' vs dKO-V' (n=3) [cell set #2]
78 (up) and 207 (down)
FDR < 0.05 & FC > 1.5
high-confidence dKO-8 DEGs
100 (up) and 303 (down)
FDR < 0.05 in all batches;
FC (batch average) > 1.5
** CDK8 excluded*
high-confidence dKO-19 DEGs
70(up) and 150 (down)
FDR < 0.05 in all batches;
FC (batch average) > 1.5
** CDK19 excluded*
high-confidence DEGs
regulated by CDK8/19
Total Number: 429
union of validated dKO-8 and
dKO-19 DEGs
B
WT_SnxB vs Ctrl_3h (n=3) [Batch #1]
0 (up) and 53 (down)
FDR < 0.05 & FC > 1.5
WT_SnxB vs Ctrl_3h (n=3) [Batch #2]
1 (up) and 55 (down)
FDR < 0.05 & FC > 1.5
WT_SnxB vs Ctrl_3h (n=3) [Batch #3]
1 (up) and 44 (down)
FDR < 0.05 & FC > 1.5
WT_SnxB vs Ctrl_24h (n=3) [Batch #1]
261 (up) and 40 (down)
FDR < 0.05 & FC > 1.5
WT_SnxB vs Ctrl_24h (n=3) [Batch #2]
200 (up) and 26 (down)
FDR < 0.05 & FC > 1.5
WT_SnxB vs Ctrl_24h (n=3) [Batch #3]
198 (up) and 40 (down)
FDR < 0.05 & FC > 1.5
WT_SnxB vs Ctrl_3d (n=3) [Batch #1]
393 (up) and 41 (down)
FDR < 0.05 & FC > 1.5
WT_SnxB vs Ctrl_3d (n=3) [Batch #2]
610 (up) and 95 (down)
FDR < 0.05 & FC > 1.5
high-confidence SnxB_3h DEGs
0 (up) and 46 (down)
FDR < 0.05 in all batches;
FC (batch average) > 1.5
high-confidence SnxB_24h DEGs
100 (up) and 23 (down)
FDR < 0.05 in all batches;
FC (batch average) > 1.5
high-confidence SnxB_3d DEGs
359 (up) and 37 (down)
FDR < 0.05 in all batches;
FC (batch average) > 1.5
high-confidence DEGs
regulated by Senexin B
Total Number: 436
union of SnxB_3h, 24h, 72h

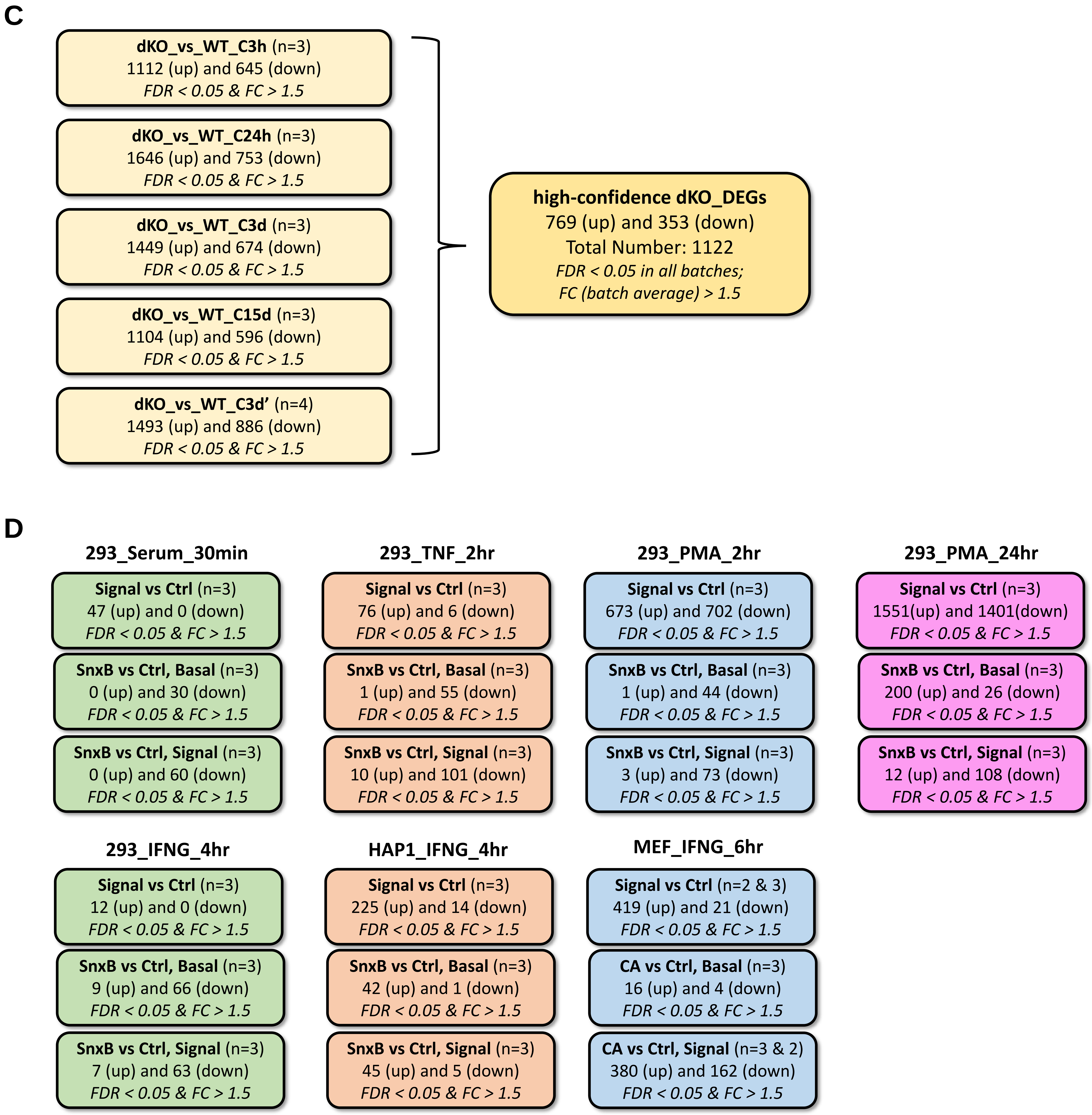

Figure S1. Schemes of identifying differentially expressed genes (DEGs).

(A) Scheme of identifying high-confidence DEGs regulated by CDK8 or CDK19 reconstitution in double-knockout (dKO) 293 cells. **(B)** Scheme of identifying high-confidence DEGs regulated by Senexin B (SnxB) in parental 293 cells. **(C)** Scheme of identifying high-confidence DEGs regulated by dKO (relative to parental WT cells) in 293. **(D)** Scheme of identifying DEGs affected by the indicated signals and CDK8/19 inhibitors.

Figure S2

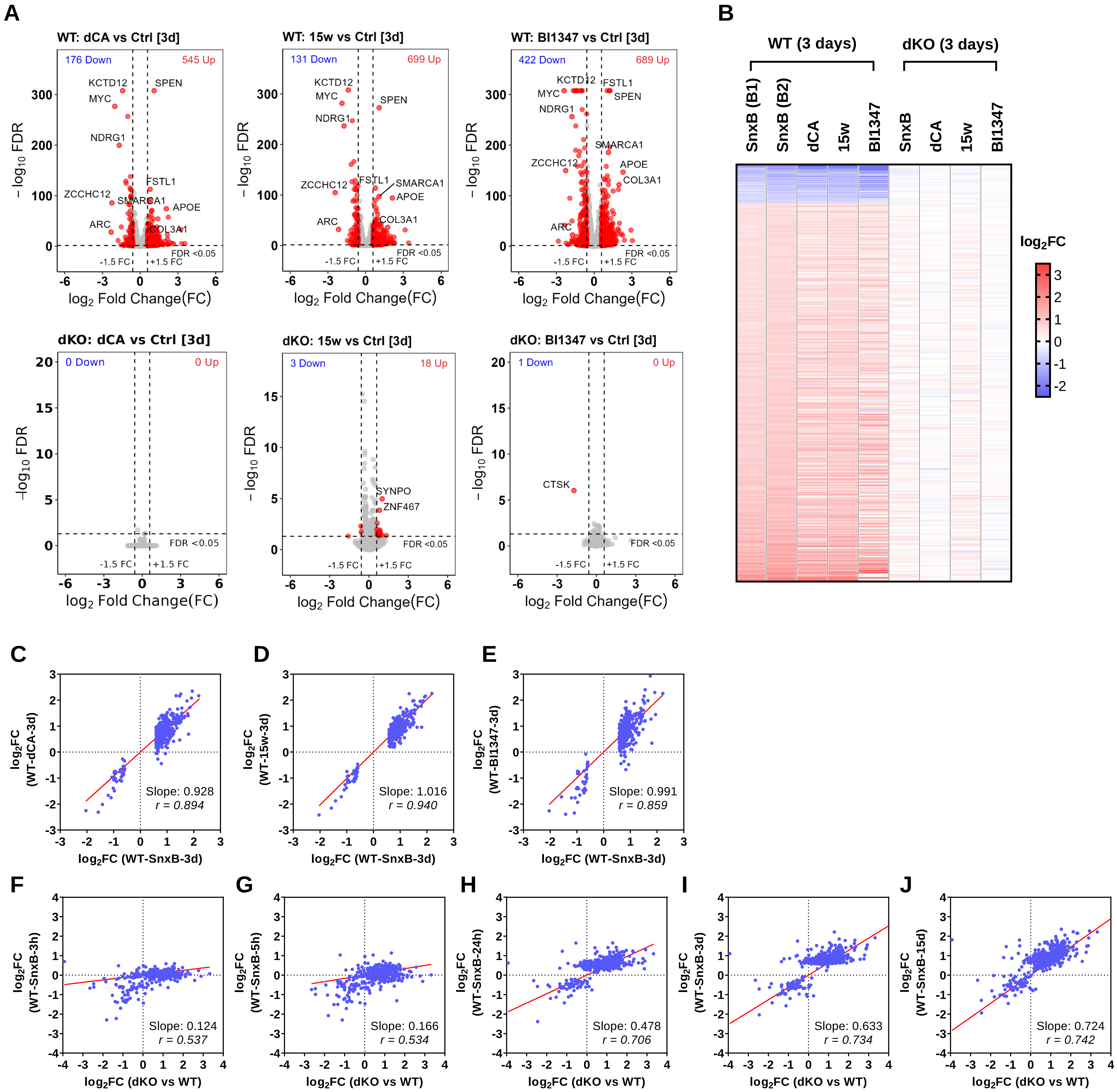

Figure S2. Transcriptomic analysis of the effects of different Mediator kinase inhibitors and CDK8/19 knockout on gene expression. **(A)** Volcano plots of the effects of 72-hr treatment with 1 µM dCA, 1 µM 15w and 200nM BI1347 on gene expression in the parental (WT) 293 cells (above) and their dKO derivative (below). **(B)** Heatmap of the effects of 3-day treatment with 1 µM Senexin B (SnxB, two independent batches), 1 µM didehydrocortistatin A (dCA), 1 µM 15w and 200nM BI1347 on 396 DEGs regulated by 3-day Senexin B treatment. **(C-E)** Comparison of the effects of 3-day treatment with dCA **(C),** 15w **(D)** and BI1347 **(E)** with the effects of Senexin B treatment (average of independent batches). Slope and Pearson correlation coefficients (r) were calculated by linear regression and correlation analysis. **(F-J)** Comparison of the effects of Senexin B treatment for 3 hrs (**F**), 5 hrs **(G)**, 24 hrs (**H**), 3 days (**I**) and 15 days (**J**) with the effects of dKO for the 436 high-confidence DEGs regulated by Senexin B. Slope and Pearson correlation coefficients (r) were calculated by linear regression and correlation analysis.

Figure S3

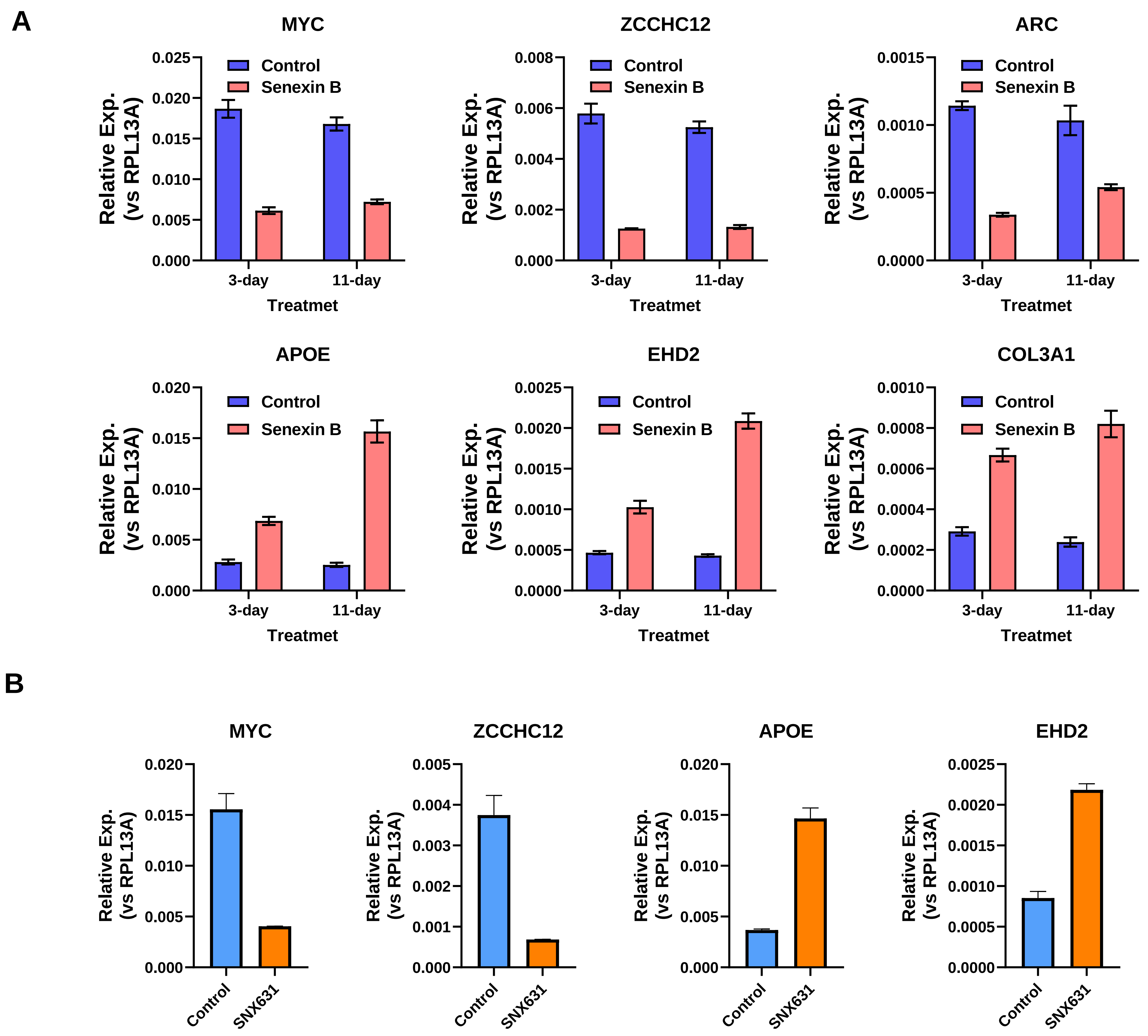

Figure S3. qPCR validation of CDK8/19 inhibitor-regulated genes identified by RNA-Seq analysis.

(A) 293 cells were cultured in vehicle control (0.1% DMSO) or 1 µM CDK8/19 inhibitor Senexin B for 3 days or 11 days before RNA extraction and qPCR analysis of mRNA expression of the indicated genes. **(B)** 293 cells were cultured in vehicle control (0.1% DMSO) or 500 nM CDK8/19 inhibitor SNX631 for 3 days before RNA extraction and qPCR analysis of mRNA expression of the indicated genes.

Figure S4

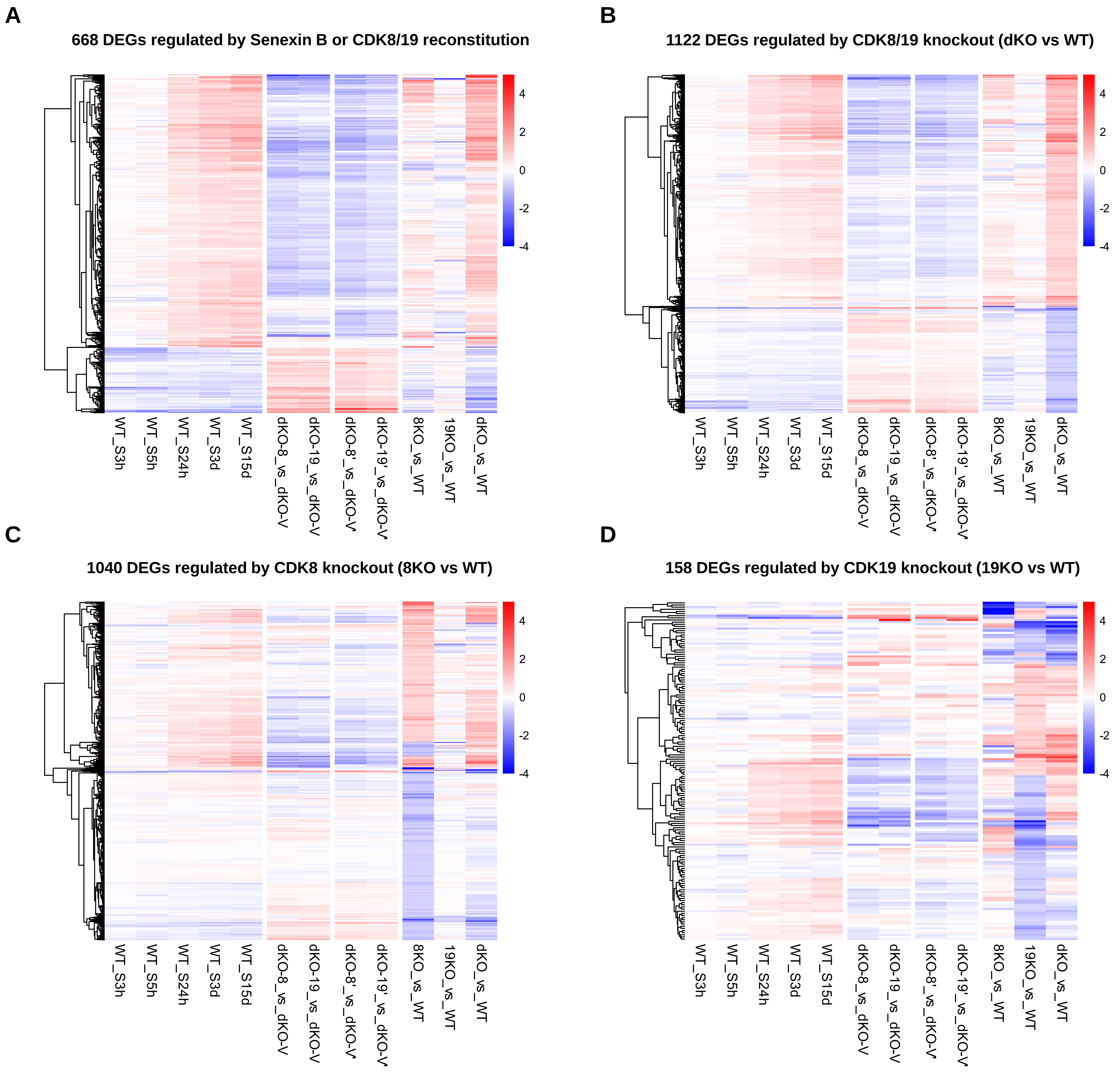

Figure S4. RNA-Seq analysis of the effects of CDK8/19 inhibition, knockout and reconstitution on the expression of DEG sets derived from different comparisons.

(A) Heatmap of the combined set of 668 DEGs regulated by CDK8/19 expression in dKO cells or by Senexin B at different time points (Fig. S1A,B) under the indicated conditions. **(B)** Heatmap of 1122 DEGs, identified by comparing dKO and WT cells (in biological triplicates, five batches, Fig. S1C) under the same conditions. **(C)** Heatmap of 1040 DEGs, identified by comparing 8KO and WT cells (in biological triplicates, single batch) under the same conditions. **(D)** Heatmap of 158 DEGs, identified by comparing 19KO and WT cells (in biological triplicates, single batch) under the same conditions.

Figure S5

A

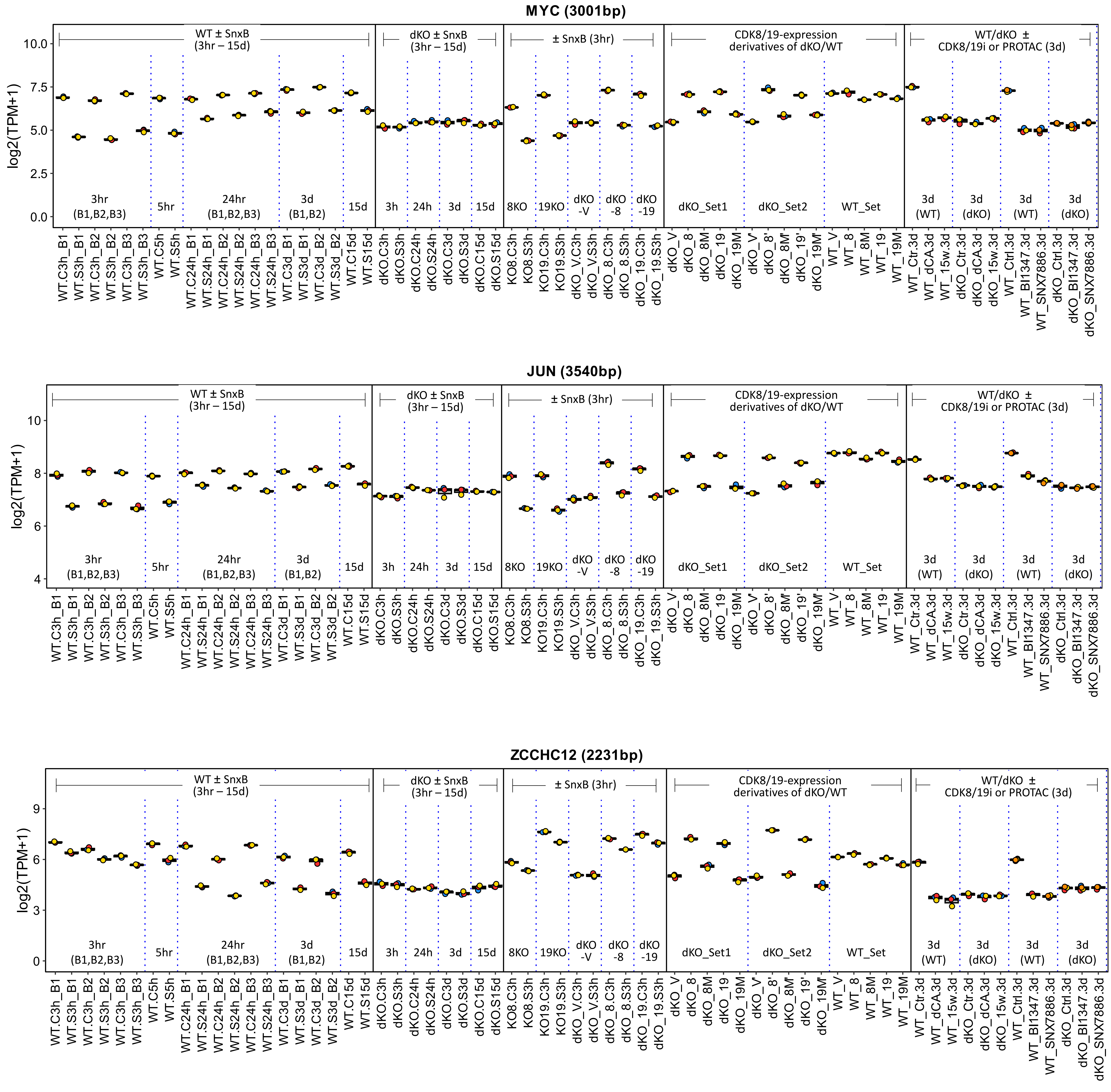

MYC (3001bp)
log2(TPM+1)
10.0
7.5
5.0
2.5
0.0
WT ± SnxB (3hr – 15d)
dKO ± SnxB (3hr – 15d)
± SnxB (3hr)
CDK8/19-expression derivatives of dKO/WT
WT/dKO ± CDK8/19i or PROTAC (3d)
3hr (B1,B2,B3)
5hr
24hr (B1,B2,B3)
3d (B1,B2)
15d
3h
24h
3d
15d
8KO
19KO
dKO -V
dKO -8
dKO -19
dKO_Set1
dKO_Set2
WT_Set
3d (WT)
3d (dKO)
3d (WT)
3d (dKO)
WT.C3h_B1
WT.S3h_B1
WT.C3h_B2
WT.S3h_B2
WT.C3h_B3
WT.S3h_B3
WT.C5h
WT.S5h
WT.C24h_B1
WT.S24h_B1
WT.C24h_B2
WT.S24h_B2
WT.C24h_B3
WT.S24h_B3
WT.C3d_B1
WT.S3d_B1
WT.C3d_B2
WT.S3d_B2
WT.C15d
WT.S15d
dKO.C3h
dKO.S3h
dKO.C24h
dKO.S24h
dKO.C3d
dKO.S3d
dKO.C15d
dKO.S15d
KO8.C3h
KO8.S3h
KO19.C3h
KO19.S3h
dKO_V.C3h
dKO_V.S3h
dKO_8.C3h
dKO_8.S3h
dKO_19.C3h
dKO_19.S3h
dKO_V
dKO_8
dKO_8M
dKO_19
dKO_19M
dKO_V'
dKO_8'
dKO_8M'
dKO_19'
dKO_19M'
WT_V
WT_8
WT_8M
WT_19
WT_19M
WT_Ctr.3d
WT_dCA.3d
WT_15w.3d
dKO_Ctr.3d
dKO_dCA.3d
dKO_15w.3d
WT_Ctrl.3d
WT_BI1347.3d
WT_SNX7886.3d
dKO_Ctrl.3d
dKO_BI1347.3d
dKO_SNX7886.3d
JUN (3540bp)
log2(TPM+1)
10
8
6
4
ZCCHC12 (2231bp)
log2(TPM+1)
9
6
3
0

B

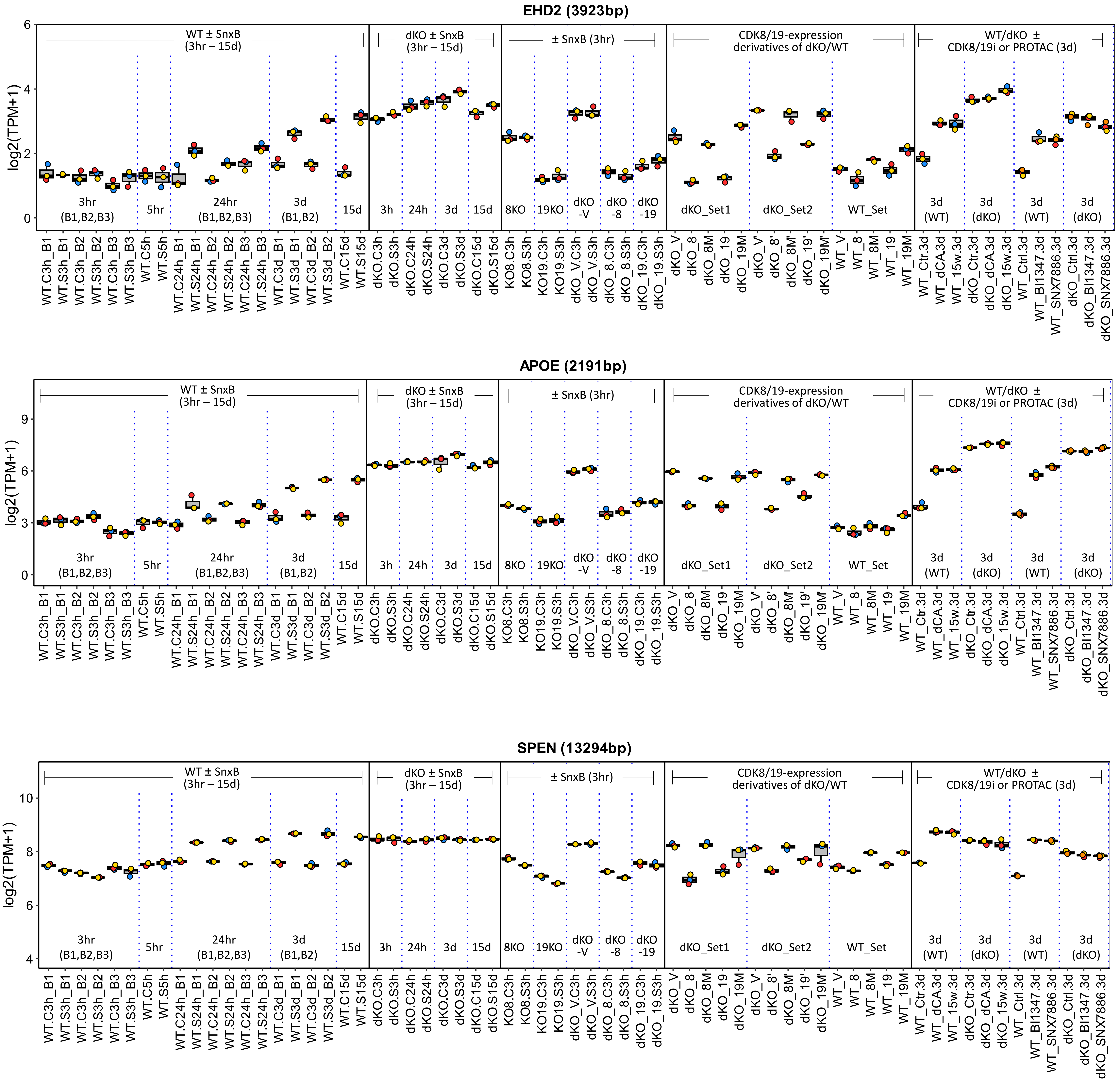
EHD2 (3923bp)
APOE (2191bp)
SPEN (13294bp)
log2(TPM+1)
WT ± SnxB (3hr – 15d)
dKO ± SnxB (3hr – 15d)
± SnxB (3hr)
CDK8/19-expression derivatives of dKO/WT
WT/dKO ± CDK8/19i or PROTAC (3d)
3hr (B1,B2,B3)
5hr
24hr (B1,B2,B3)
3d (B1,B2)
15d
3h
24h
3d
15d
8KO
19KO
dKO -V
dKO -8
dKO -19
dKO_Set1
dKO_Set2
WT_Set
3d (WT)
3d (dKO)
WT.C3h_B1
WT.S3h_B1
WT.C3h_B2
WT.S3h_B2
WT.C3h_B3
WT.S3h_B3
WT.C5h
WT.S5h
WT.C24h_B1
WT.S24h_B1
WT.C24h_B2
WT.S24h_B2
WT.C24h_B3
WT.S24h_B3
WT.C3d_B1
WT.S3d_B1
WT.C3d_B2
WT.S3d_B2
WT.C15d
WT.S15d
dKO.C3h
dKO.S3h
dKO.C24h
dKO.S24h
dKO.C3d
dKO.S3d
dKO.C15d
dKO.S15d
KO8.C3h
KO8.S3h
KO19.C3h
KO19.S3h
dKO_V.C3h
dKO_V.S3h
dKO_8.C3h
dKO_8.S3h
dKO_19.C3h
dKO_19.S3h
dKO_V
dKO_8
dKO_8M
dKO_19
dKO_19M
dKO_V'
dKO_8'
dKO_8M'
dKO_19'
dKO_19M'
WT_V
WT_8
WT_8M
WT_19
WT_19M
WT_Ctr.3d
WT_dCA.3d
WT_15w.3d
dKO_Ctr.3d
dKO_dCA.3d
dKO_15w.3d
WT_Ctrl.3d
WT_BI1347.3d
WT_SNX7886.3d
dKO_Ctrl.3d
dKO_BI1347.3d
dKO_SNX7886.3d

C

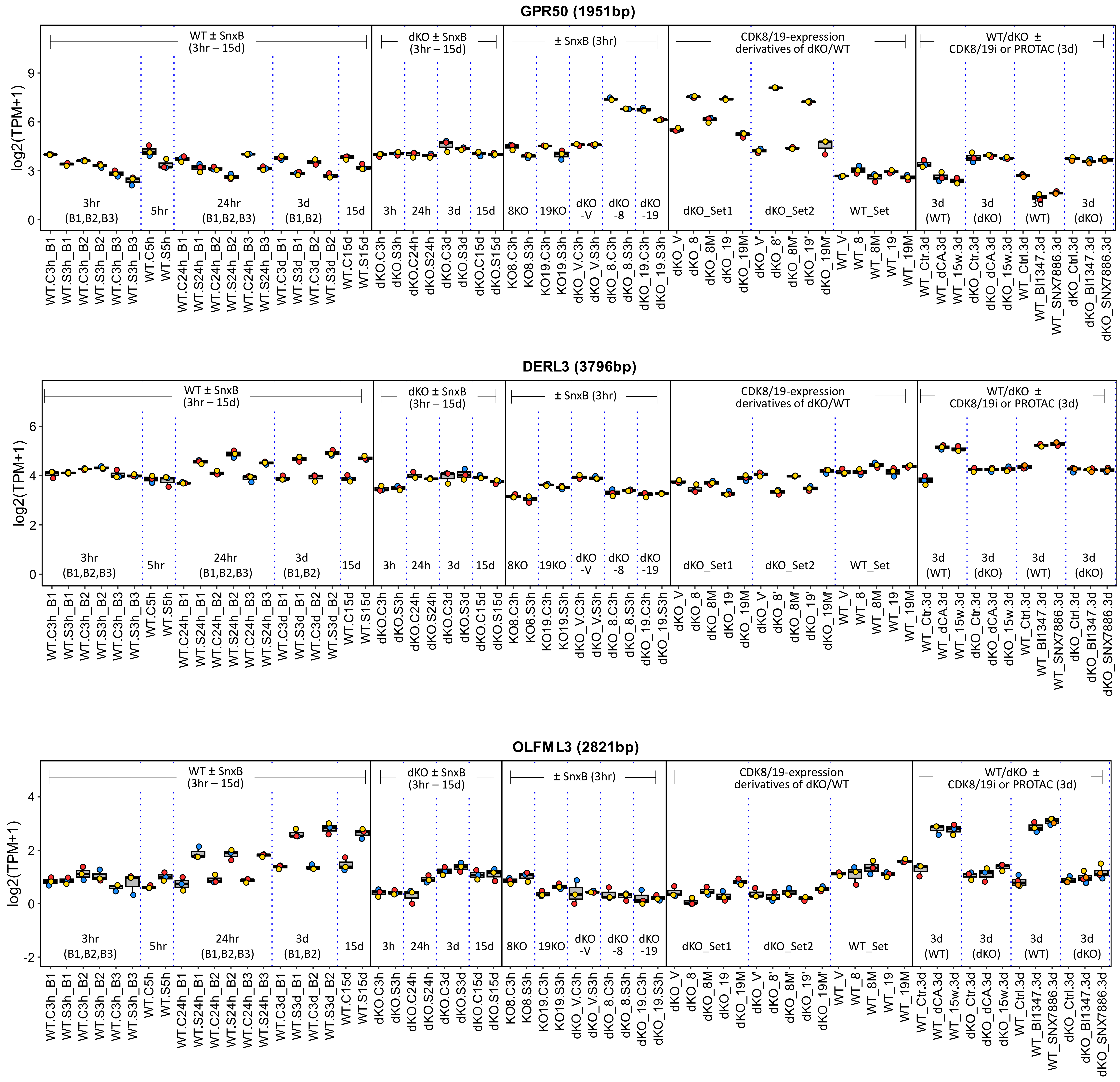
GPR50 (1951bp)
DERL3 (3796bp)
OLFML3 (2821bp)
log2(TPM+1)
WT ± SnxB (3hr – 15d)
dKO ± SnxB (3hr – 15d)
± SnxB (3hr)
CDK8/19-expression derivatives of dKO/WT
WT/dKO ± CDK8/19i or PROTAC (3d)
3hr (B1,B2,B3)
5hr
24hr (B1,B2,B3)
3d (B1,B2)
15d
3h
24h
3d
15d
8KO
19KO
dKO -V
dKO -8
dKO -19
dKO_Set1
dKO_Set2
WT_Set
3d (WT)
3d (dKO)
WT.C3h_B1
WT.S3h_B1
WT.C3h_B2
WT.S3h_B2
WT.C3h_B3
WT.S3h_B3
WT.C5h
WT.S5h
WT.C24h_B1
WT.S24h_B1
WT.C24h_B2
WT.S24h_B2
WT.C24h_B3
WT.S24h_B3
WT.C3d_B1
WT.S3d_B1
WT.C3d_B2
WT.S3d_B2
WT.C15d
WT.S15d
dKO.C3h
dKO.S3h
dKO.C24h
dKO.S24h
dKO.C3d
dKO.S3d
dKO.C15d
dKO.S15d
KO8.C3h
KO8.S3h
KO19.C3h
KO19.S3h
dKO_V.C3h
dKO_V.S3h
dKO_8.C3h
dKO_8.S3h
dKO_19.C3h
dKO_19.S3h
dKO_V
dKO_8
dKO_8M
dKO_19
dKO_19M
dKO_V'
dKO_8'
dKO_8M'
dKO_19'
dKO_19M'
WT_V
WT_8
WT_8M
WT_19
WT_19M
WT_Ctr.3d
WT_dCA.3d
WT_15w.3d
dKO_Ctr.3d
dKO_dCA.3d
dKO_15w.3d
WT_Ctrl.3d
WT_BI1347.3d
WT_SNX7886.3d
dKO_Ctrl.3d
dKO_BI1347.3d
dKO_SNX7886.3d

D

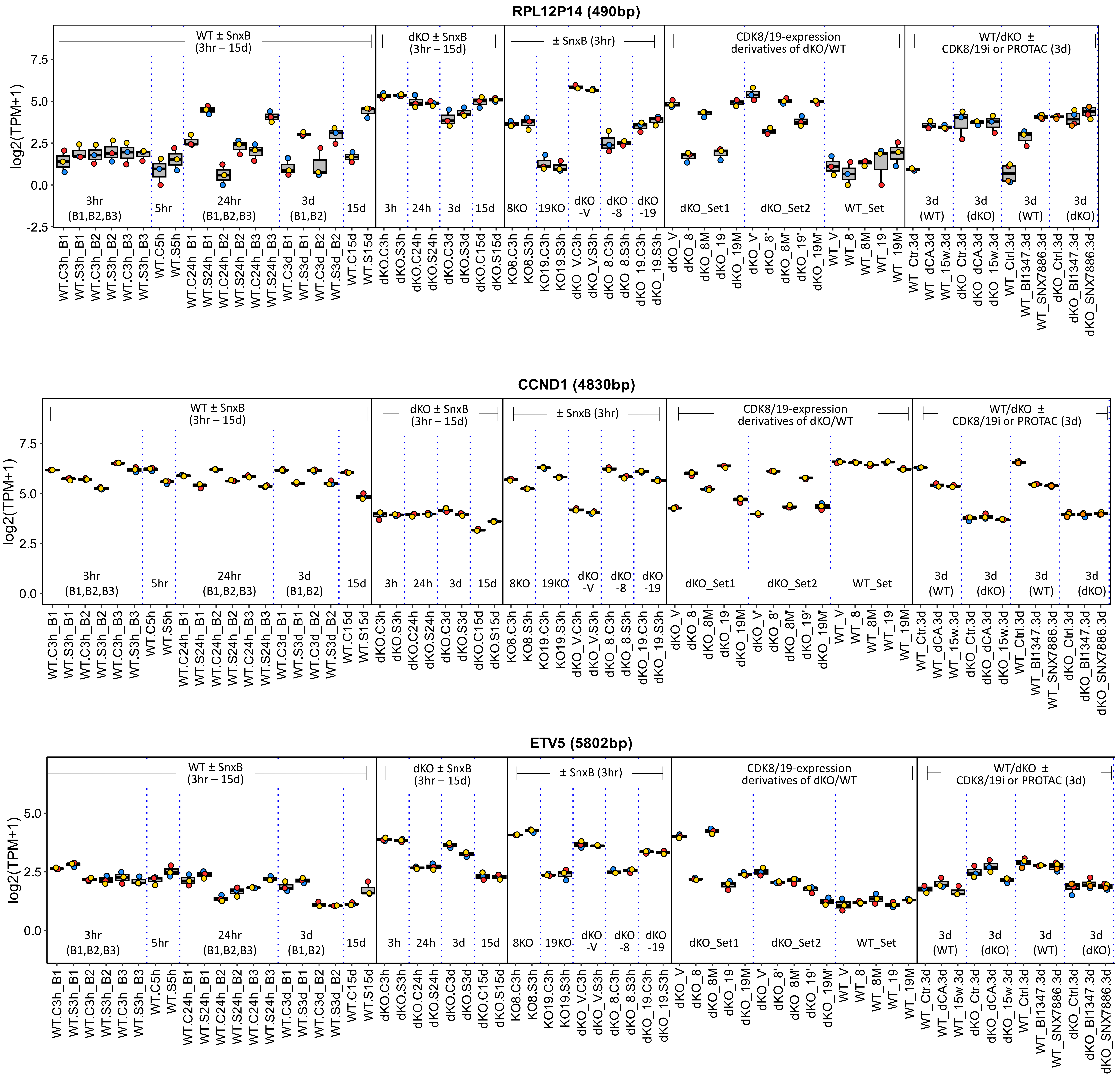

Figure S5. Variation in the expression of representative CDK8/19-regulated genes under different conditions (RNA-Seq, TPM). **(A)** Genes positively regulated by CDK8/19. **(B)** Genes negatively regulated by CDK8/19. **(C)** Genes regulated by CDK8/19 inhibition but not by dKO. **(D)** Genes showing variable response to CDK8/19 mutants or inhibitors in different batches.

Figure S6

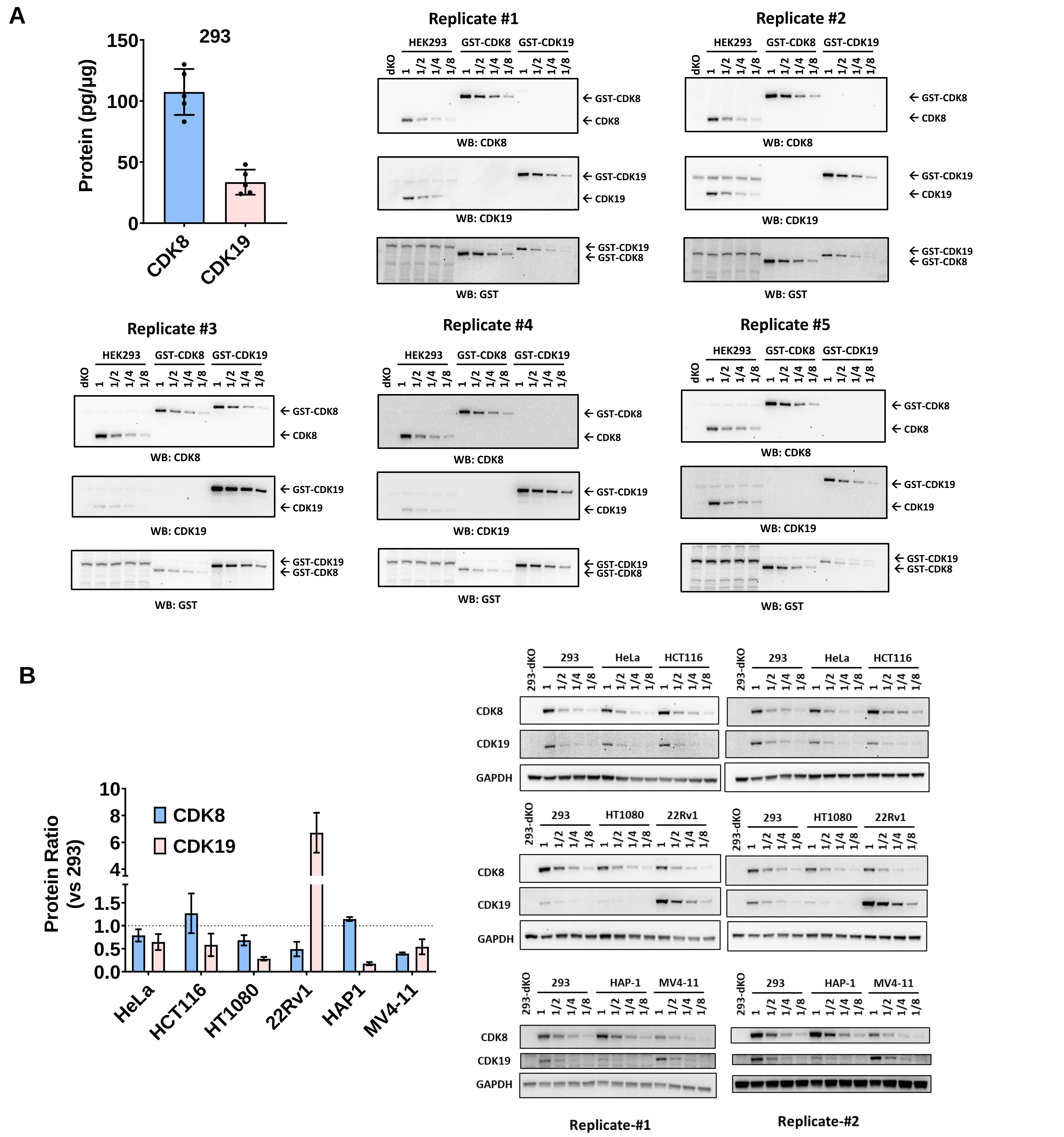

Figure S6. Stoichiometry of CDK8 and CDK19 proteins in different cell lines.

(A) Quantitative immunoblotting analysis of the ratio of CDK8 to CDK19 proteins in 293 cells. Serially diluted whole cell extract (starting from 25 µg) and serially diluted GST-CDK8 and GST-CDK19 recombinant proteins (starting from 10 ng) were loaded on the same SDS-PAGE. 293-dKO whole cell extract was used as negative control and mixed with test samples to adjust the loading to the same amount of total protein. The replicates shown here and used for protein ratio calculations are those where signals of diluted cell extracts fall in the linear range of the standard curve of the recombinant proteins. **(B)** Quantitative immunoblotting analysis of CDK8 and CDK19 proteins in different cell lines. Serially diluted whole cell extracts of HeLa, HCT116, HT1080, HAP1, MV4-11 and 22Rv1 cells (starting from 25 µg) were run in parallel with serially diluted 293 whole cell extracts. CDK8 and CDK19 protein expression levels in these cell lines were normalized to CDK8/19 protein levels in 293 cells.
